# Supplementary material for: A group B Streptococcus alpha-like protein subunit vaccine induces functionally active antibodies in humans targeting homotypic and heterotypic strains
Source: Cell Rep Med. 2022 Feb 15;3(2):100511. doi: 10.1016/j.xcrm.2022.100511 (PMC8861819; doi:10.1016/j.xcrm.2022.100511)
Supplement: Document S1. Tables S1–S3 [file mmc1.pdf]

Cell Reports Medicine, Volume *3*

Supplemental information

A group B *Streptococcus* alpha-like protein subunit vaccine induces functionally active antibodies in humans targeting homotypic and heterotypic strains

Andrzej Pawlowski, Jonas Lannergård, Majela Gonzalez-Miro, Duojia Cao, Sara Larsson, Jenny J. Persson, Geoff Kitson, Michael Darsley, Ane Lilleøre Rom, Morten Hedegaard, Per B. Fischer, and Bengt Johansson-Lindbom

Table S1. Alp-N domain specific serum IgG, IgA and IgM geometric mean concentrations (GMC) and fold-increases relative pre-vaccination baseline levels (Day 0) four weeks after the second vaccine dose (Day 57). The two separate cohorts receiving 50 µg GBS-NN plus AlOH in the clinical phase IA (n=8) and 1B (n=45) studies are included in the analyses (n=53 in total). Related to Figures 1 and 2.

	**IgG GMC**			**IgA GMC**			**IgM GMC**		
	(µg/ml) (95% CI)		Fold-increase	(µg/ml) (95% CI)		Fold-increase	(µg/ml) (95% CI)		Fold-increase
	Day 0	Day 57	(95% CI)	Day 0	Day 57	(95% CI)	Day 0	Day 57	(95% CI)
αC-N	**0.12**	**5.45**	**44**	**0.15**	**2.23**	**15**	**0.89**	**1.65**	**1.9**
	(0.09 - 0.16)	(3.71 - 8.01)	(32 - 62)	(0.11 - 0.21)	(1.35 - 3.69)	(10 - 21)	(0.70 - 1.13)	(1.34 - 2.04)	(1.4 - 2.4)
Rib-N	**0.05**	**2.22**	**42**	**0.09**	**0.91**	**10**	**1.16**	**1.42**	**1.2**
	(0.04 - 0.06)	(1.67 - 2.96)	(31 - 57)	(0.07 - 0.11)	(0.61 - 1.35)	(7.6 - 14)	(0.96 - 1.38)	(1.12 - 1.80)	(1.0 - 1.5)
Alp1-N	**0.14**	**1.15**	**8.1**	**0.17**	**0.56**	**3.4**	nd	nd	-
	(0.11 - 0.18)	(0.71 - 1.84)	(5.8 - 11)	(0.12 - 0.22)	(0.35 - 0.89)	(2.5 - 4.6)	-	-	-
Alp2/3-N	**0.21**	**2.42**	**11**	**0.13**	**1.04**	**8.1**	nd	nd	-
	(0.16 - 0.28)	(1.52 - 3.86)	(8.0 - 16)	(0.09 - 0.17)	(0.66 - 1.64)	(6.0 - 11)	-	-	-

Table S2. Vaccine-induced OPk (ΔOPkA titers) of clinical isolates possessing the αC gene and collected from cases of EOD (reference 34) as determined with pre- and post-vaccination sera from two vaccinees achieving high levels of αC-specific IgG. Related to Figure 4.

Serum pair[a]	GBS isolate	Invasive disease	Capsule type	ΔOPkA titer[d]	TR A909[e]
A03[b]	1001	EOD	Ia	2050	1.93
	1044	EOD	V	3430	3.27
	1047	EOD	Ib	1900	n.d.
	1084	EOD	II	3630	6.05
B10[c]	1001	EOD	Ia	6015	1.99
	1044	EOD	V	8880	3.36
	1047	EOD	Ib	16700	n.d.
	1084	EOD	II	10925	7.09

[a] day 0 and day 57 post vaccination sera
[b] αC-specific IgG conc. were 0.37 and 42 µg/ml for day 0 and day 57, respectively
[c] αC-specific IgG conc. were 0.67 and 93 µg/ml for day 0 and day 57, respectively
[d] vaccine-induced OPkA titer = titer (day 57) - titer (day 0)
[e] Relative titer = ΔOPkA titer for isolate / ΔOPkA titer for strain A909
n.d. ΔOPkA titer for strain A909 not determined in experiment

Table S3. Pre- and post-vaccination (day 0 versus day 57) concentration of antibodies against αC-N and Rib-N, as well as vaccine-induced OPk titers (ΔOPkA titer) against A909 and BM110, for the selected sera studied in Figure 5A. Related to Figure 5. For results presented in Figure 5A, all sera were assayed at a fixed dilution of 1/100.

Subject	αC-N IgG (μg/ml)		αC-N IgA (μg/ml)		Rib-N IgG (μg/ml)		Rib-N IgA (μg/ml)		ΔOPkA titer	
	Day 0	Day 57	Day 0	Day 57	Day 0	Day 57	Day 0	Day 57	A909	BM110
A02	0.10	4.85	0.10	3.82	0.09	5.33	0.12	1.99	1100	380
A03	0.37	42.4	1.16	36.9	0.04	5.61	0.16	2.80	8445	210
A06	0.16	42.0	0.08	10.8	0.02	2.47	0.08	1.73	9690	45
A08	0.21	27.0	1.31	15.3	0.04	7.54	0.12	2.02	5880	310
