# Supplementary material for: A group B Streptococcus alpha-like protein subunit vaccine induces functionally active antibodies in humans targeting homotypic and heterotypic strains
Source: Cell Rep Med. 2022 Feb 15;3(2):100511. doi: 10.1016/j.xcrm.2022.100511 (PMC8861819; doi:10.1016/j.xcrm.2022.100511)
Supplement: Document S2. Article plus supplemental information [file mmc2.pdf]

Cell Reports
Medicine

Article

A group B *Streptococcus* alpha-like protein subunit vaccine induces functionally active antibodies in humans targeting homotypic and heterotypic strains

Graphical abstract

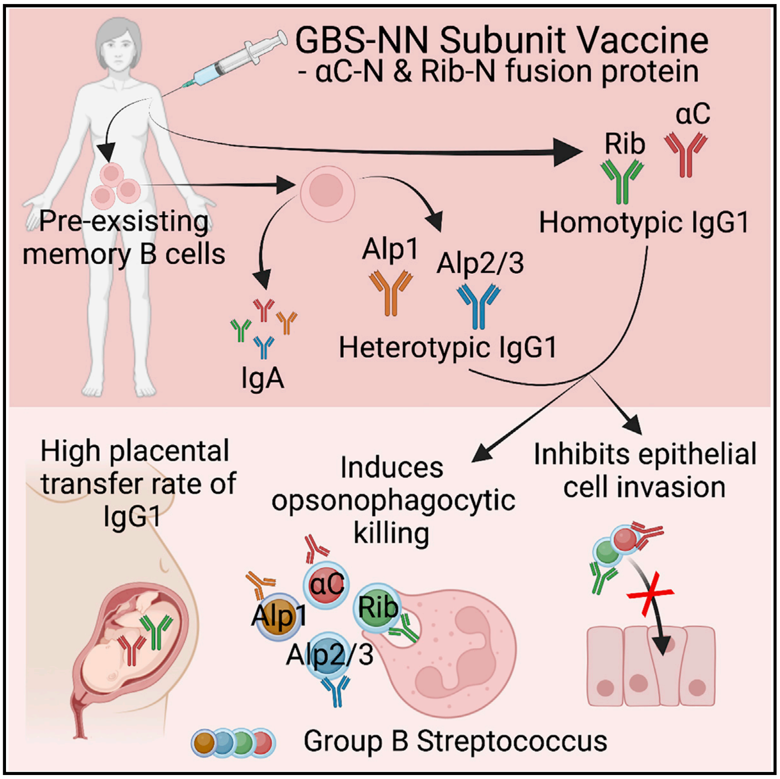

Highlights

- GBS-NN subunit vaccine broadly elicits IgG1 to homotypic αC and Rib N-terminal domains
- IgA and heterotypic IgG responses occur in vaccinees with pre-existing immunity
- Abs mediate opsonophagocytic killing and prevent bacterial epithelial cell invasion
- IgG against αC-N and Rib-N is transferred efficiently across the placenta

Authors

Andrzej Pawlowski, Jonas Lannergård, Majela Gonzalez-Miro, ..., Morten Hedegaard, Per B. Fischer, Bengt Johansson-Lindbom

Correspondence

bengt.johansson_lindbom@med.lu.se

In brief

GBS is a leading cause of neonatal infections. A maternal vaccine is prioritized by the WHO. Pawlowski et al. show that an alpha-like protein subunit vaccine elicits placentally transferable IgG1 and IgA. The antibodies mediate opsonophagocytic killing of vaccine homotypic and heterotypic strains and prevent bacterial invasion of epithelial cells.

Pawlowski et al., 2022, Cell Reports Medicine *3*, 100511
February 15, 2022 © 2022 The Author(s).
https://doi.org/10.1016/j.xcrm.2022.100511

CellPress

Cell Reports Medicine

CellPress
OPEN ACCESS

Article

A group B *Streptococcus* alpha-like protein subunit vaccine induces functionally active antibodies in humans targeting homotypic and heterotypic strains

Andrzej Pawlowski,[1] Jonas Lannergård,[1] Majela Gonzalez-Miro,[1] Duojia Cao,[1] Sara Larsson,[1] Jenny J. Persson,[1] Geoff Kitson,[2] Michael Darsley,[2] Ane Lilleøre Rom,[3,4] Morten Hedegaard,[3] Per B. Fischer,[2] and Bengt Johansson-Lindbom[1,2,5,*]

[1]Immunology Section, BMC D14, Lund University, 221 84 Lund, Sweden
[2]Minervax A/S, Ole Maaløes Vej 3, 2200 Copenhagen N, Denmark
[3]Department of Obstetrics, the Juliane Marie Centre, Copenhagen University Hospital Rigshospitalet, 2100 Copenhagen, Denmark
[4]The Research Unit for Women's and Children's Health, the Juliane Marie Centre, Copenhagen University Hospital Rigshospitalet, 2100 Copenhagen, Denmark
[5]Lead contact
*Correspondence: bengt.johansson_lindbom@med.lu.se
https://doi.org/10.1016/j.xcrm.2022.100511

SUMMARY

Maternal vaccination is a promising strategy for preventing neonatal disease caused by group B *Streptococcus*. The safety and immunogenicity of the prototype vaccine GBS-NN, a fusion protein consisting of the N-terminal domains of the alpha-like proteins (Alp) αC and Rib, were recently evaluated favorably in healthy adult women in a phase 1 trial. Here we demonstrate robust immunoglobulin G (IgG) and immunoglobulin A (IgA) responses against αC and Rib, as well as against the heterotypic Alp family members Alp1–Alp3. IgA and heterotypic IgG responses are more variable between subjects and correlate with pre-existing immunity. Vaccine-induced IgG mediates opsonophagocytic killing and prevents bacterial invasion of epithelial cells. Like the vaccine-induced response, naturally acquired IgG against the vaccine domains is dominated by IgG1. Consistent with the high IgG1 cross-placental transfer rate, naturally acquired IgG against both domains reaches higher concentrations in neonatal than maternal blood, as assessed in a separate group of non-vaccinated pregnant women and their babies.

INTRODUCTION

Group B *Streptococcus* (GBS; *Streptococcus agalactiae*) is an encapsulated Gram-positive bacterium that colonizes the gastrointestinal mucosa and female genital tract. Estimations suggest that 11%–35% of all women worldwide are colonized, with the wide range reflecting regional variations.[1,2] GBS is an opportunistic pathogen and the leading cause of life-threatening infections in newborns, with meningitis, pneumonia, and sepsis as the main clinical manifestations.[3] Neonatal infections appear as early-onset disease (EOD) during the first 7 days of life or late-onset disease (LOD) from 8 days to 3 months after birth. In addition, GBS is associated with ascending infections and chorioamnionitis in pregnant women, which can cause stillbirth and preterm delivery.[3–6] Although the incidence of these GBS-related adverse pregnancy outcomes is not fully appreciated, it has been estimated that GBS underlies 1%–4% of all stillbirths, with the highest incidence in Africa.[4] In some high-income countries, screening and/or identification of clinical risk factors, coupled with intrapartum antibiotic prophylaxis (IAP), has reduced the incidence of EOD[7] but has had no effect on LOD.[8] In addition, IAP has no effect on adverse pregnancy outcomes and has not been implemented as a routine practice in low- and middle-income countries. Currently, no approved GBS vaccine exists; development of a maternal GBS vaccine has been identified as a key priority by the World Health Organization (WHO).[9]

We have recently reported that the prototype subunit vaccine GBS-NN displays good safety and immunogenicity profiles in a randomized, placebo-controlled, double-blind phase I study involving 240 vaccinated adult healthy women.[10] GBS-NN is a fusion protein consisting of the N-terminal domains of the GBS proteins AlphaC (αC) and Rib,[11] members of the alpha-like protein (Alp) family also containing Alp1–Alp4.[12] The Alps are allelic variants, and at least one Alp was detected in 99.3% of 6,340 sequenced invasive GBS isolates collected in the United States during the period of 2015–2017; Alp4 was not detected in any of these isolates.[13] The Alps are cell surface proteins with a C-terminal domain containing a cell wall-anchoring motif, varying numbers of repeat regions, and an N-terminal domain protruding from the polysaccharide capsule.[12] Individual Alps show preference for certain capsular polysaccharides (CPS) types, with the strongest association shown for Rib and CPS III.[12,13] Nonetheless, immunization of mice with purified Rib confers protection

This is an open access article under the CC BY license (http://creativecommons.org/licenses/by/4.0/).

against GBS strains possessing the Rib gene combined with serotypes distinct from type III,[14,15] indicating that Rib is accessible for protective antibodies (Abs) irrespective of associated CPS type. The extracellular exposure, combined with the exceptionally broad coverage of clinical isolates, makes Alp N-terminal domains (Alp-Ns) highly relevant as vaccine candidates. Because Alp2-N and Alp3-N have the same amino acid sequence,[16] an efficacious GBS vaccine based on αC-N, Rib-N, Alp1-N, and Alp2/3-N would provide protection against essentially all invasive GBS strains. Because all N-terminal domains display a high degree of sequence homology,[16] our original hypothesis was that broad coverage could be achieved with a vaccine consisting of only αC-N and Rib-N. In the current study, we assessed the protective functions of the vaccine response in relation to vaccine homotypic and heterotypic GBS strains.

Intraamniotic inoculation of GBS in a primate model leads to bacterial localization within neonatal alveolar epithelial cells, demonstrating a possible mechanism for GBS dissemination in the neonate.[17] Likewise, GBS adheres to and transcytoses intact human chorion epithelial cell monolayers and can be cultured from the chorioamniotic membrane of women affected by premature labor,[18–20] indicating that cellular invasion is a mechanism for GBS to cross epithelial barriers and cause ascending infection in pregnant women. Invasion by GBS has similarly been demonstrated for a number of different transformed human cell lines of epithelial, brain microvascular endothelial, and myeloid origin.[21–25] The human cervical carcinoma epithelial cell line ME180 is a well-established model for studying streptococcal adherence and invasion.[25–28] αC mediates entry of GBS into ME180 cells, and this process is dependent on the N-terminal domain, but not the repeat regions,[25] and occurs through interactions with the $\alpha_1\beta_1$ integrin and glycosaminoglycan on host cells.[26,29] Additionally, a polyclonal rabbit serum against GBS-NN prevents invasion by the αC-expressing strain A909 and the Rib-expressing strain BM110.[11] These studies suggest that targeting Alp-Ns through vaccination could provide a means to interfere with GBS invasion across the epithelium, with possible effects on neonatal disease and maternal ascending infections. However, it remains to be determined whether Abs elicited in humans following GBS-NN vaccination prevent invasion of epithelial cells.

Retrospective case control studies have demonstrated inverse correlations between levels of placentally transferred GBS Abs and neonatal invasive disease.[30,31] Although these studies indicate that seroprotective thresholds might be defined, correlates of protection against neonatal GBS disease are still lacking. Assays evaluating *in vitro* surrogates of protection are therefore important for functional interpretation of phase I and II immunogenicity data before phase III efficacy studies are conducted. Protection against Gram-positive encapsulated bacteria, including *Streptococcus pneumoniae* and GBS, is dependent on Abs that mediate complement-dependent opsonophagocytic killing (OPk) by neutrophils. The ability of Abs to mediate OPk can be measured *in vitro* in the OPk assay (OPkA). This assay has been used extensively for evaluating vaccine responses against pneumococcal glycoconjugate vaccines and has greatly accelerated development and licensure of new higher valency vaccines.[32] Although GBS-NN has been shown to induce protective immunity in mice,[11] whether it is able to elicit opsonophagocytic immunoglobulin G (IgG) responses in humans is unknown.

In the current study, we focused on the cohorts from the phase I clinical study receiving two doses of 50 μg GBS-NN plus aluminum hydroxide (AlOH), a regimen found to induce optimal Ab responses to GBS-NN.[10] Our results provide a functional characterization of the Ab response in relation to vaccine homotypic and heterotypic GBS strains and demonstrate that vaccination induces robust OPk responses and an enhanced ability of serum Abs to prevent bacterial invasion of epithelial cells. In a separate study cohort with paired non-vaccinated pregnant women and neonates, we show that naturally acquired Alp-N Abs accumulate in the neonates and persist in the neonatal blood with a half-life of ~40 days.

RESULTS

GBS-NN elicits IgG responses against vaccine homotypic and heterotypic Alp-Ns

As reported previously, the clinical phase I trial of GBS-NN was divided into separate part 1A and 1B studies, representing a dose escalation and a dose confirmation study, respectively.[10] Both parts included a cohort receiving two doses of 50 μg GBS-NN plus AlOH. Although the part A cohort (n = 8) was monitored for 85 days after the first dose, in part B, blood was collected up to 1 year after vaccination (n = 45). We first set out to study long-term Ab responses against the individual Alp-N domains, focusing on the part B cohort only. Analysis of Alp-N-specific IgG 4 weeks after primary immunization (day 29) revealed significantly increased geometric mean concentrations (GMC) relative to pre-vaccination levels (Figure 1A). Increased GMCs were evident not only for the homotypic vaccine domains αC-N and Rib-N but also for IgG against the heterotypic Alp1-N and Alp2/3-N antigens ($p < 0.0001$ for all responses, one-way ANOVA). A second dose resulted in a further increase in IgG against all four Alp-Ns, and the IgG GMCs remained significantly elevated above baseline for the whole 1-year duration of the study ($p < 0.0001$ for all Alp-N domains and all time points, one-way ANOVA).

Next we focused on the peak response 4 weeks after the second dose (day 57) and here included all part A and B subjects (n = 53; Table S1). The highest IgG GMC was observed for αC-N, reaching 5.45 μg/mL. The corresponding value for Rib-N was 2.22 μg/mL. The IgG response against Alp2/3-N was comparable with that against Rib-N and reached 2.42 μg/mL, significantly higher than the response against Alp1-N, which reached an IgG GMC of 1.15 μg/mL ($p < 0.0001$). Altogether, the results show that vaccination with GBS-NN leads to persistent IgG responses not only against αC-N and Rib-N but also to heterotypic Alp1-N and Alp2/3-N, and all of these responses are boosted by a second dose.

IgG responses against vaccine heterotypic Alp-Ns depend on pre-existing immunity

All study subjects had measurable concentrations of pre-existing IgG against all four Alp-Ns, with GMCs in the order Alp2/3-N > Alp1-N > αC-N > Rib-N (Table S1). The naturally acquired

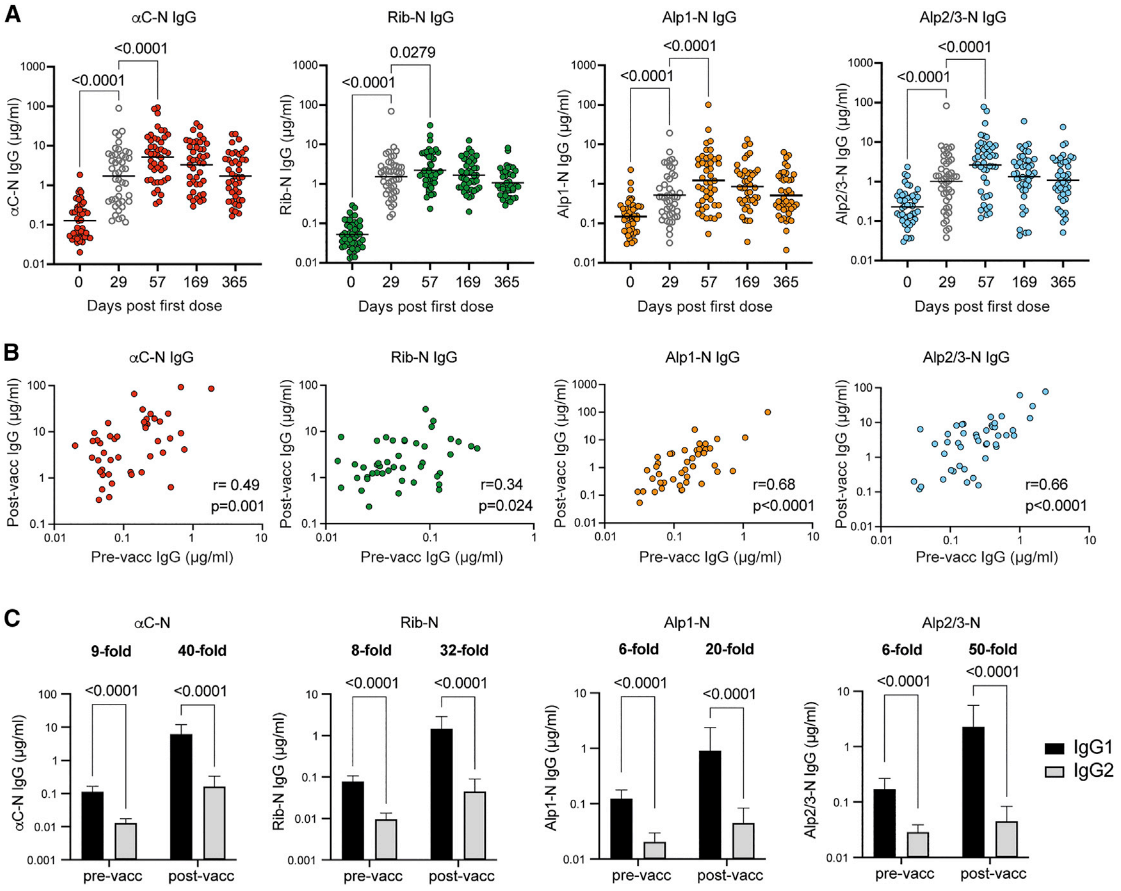

Figure 1. Vaccination with GBS-NN elicits persistent IgG responses against homotypic and heterotypic Alp-Ns

(A) Serum concentration of IgG against the indicated Alp-N before vaccination and on the indicated days after the first dose. Gray symbols represent results obtained 4 weeks after primary immunization and prior to the second dose. Results show individual subject concentrations and GMC (n = 45).

(B) Pearson correlations between pre- and post-vaccination (day 57) IgG against indicated Alp-N (n = 45).

(C) Serum concentration of IgG1 and IgG2 against the indicated Alp-N before vaccination and 4 weeks after the second dose (day 57). Results show GMCs with 95% CI (n = 20).

See also Table S1.

Ab response against the Alp-Ns was thus dominated by IgG against the heterotypic domains rather than the ones present in the vaccine. We have recently reported that the vaccine-induced IgG response against intact GBS-NN correlates with levels of pre-existing anti-GBS-NN Abs.[10] Although IgG responses against αC-N and Rib-N after two doses correlated only weakly with their homologous pre-existing IgG levels, stronger correlations between pre-and post-vaccination IgG levels were observed for the heterotypic Alp-Ns (Figure 1B). The results show that subjects exhibiting low pre-existing immunity against Alp1-N and Alp2/3-N fail to generate robust IgG responses to these domains following vaccination with GBS-NN. This was also reflected by a lower fold change relative to baseline for the heterotypic responses. Although IgG responses against αC-N and Rib-N had increased ~40-fold 4 weeks after the second dose, the levels against Alp1-N and Alp2/3-N had only increased ~10-fold at this time point (Table S1). The results suggest that IgG responses against Alp1-N and Alp2/3-N are dependent on homologous pre-existing immunity.

Naturally acquired and vaccine-induced Alp-N-specific IgG is dominated by the IgG1 subclass

GBS-NN is a candidate for maternal vaccination and therefore must stimulate production of Abs that efficiently cross the placenta. Only IgG is transferred placentally, and among the IgG subclasses IgG1 displays the highest transfer rate, whereas IgG2 crosses the placenta relatively poorly.[33] As shown in Figure 1C, naturally acquired IgG against all individual Alp-Ns was strongly dominated by IgG1, with very little contribution of IgG2. The IgG1-to-IgG2 ratio was even higher after vaccination, with IgG1 concentrations being 30–40 times higher than corresponding IgG2 levels for the homotypic N-terminal domains.

Primary GBS-NN immunization elicits IgA responses against all Alp-Ns, which correlate with pre-existing IgA levels

Like IgG, all subjects had measurable concentrations of IgA against all Alp-Ns prior to vaccination (Figure 2A). There was a significant increase in IgA GMCs against all Alp-Ns 4 weeks after

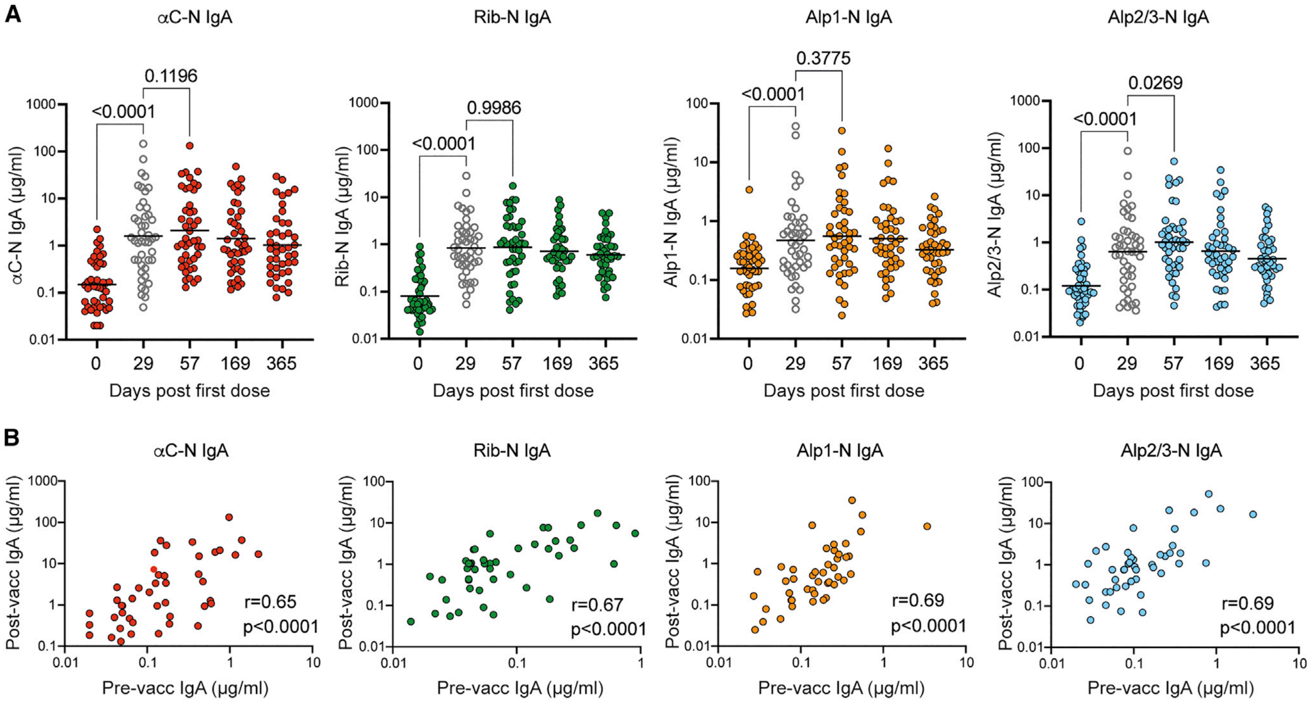

Figure 2. Primary immunization with GBS-NN elicits persistent IgA responses against homotypic and heterotypic Alp-Ns

(A) Serum concentration of IgA against the indicated Alp-N before vaccination and on the indicated days after the first dose. Gray symbols represent results obtained 4 weeks after primary immunization and prior to the second dose. Results show individual subject concentrations and GMC (n = 45).

(B) Pearson correlations between pre- and post-vaccination (day 57) IgA against the indicated Alp-N (n = 45).

See also Table S1.

primary immunization, and the GMCs then remained significantly elevated above baseline for the 1-year duration of the part B study ($p < 0.0001$ for all post-vaccination time points, one-way ANOVA). However, IgA responses elicited by primary GBS-NN immunization were only poorly (Alp2/3-N) or not at all (αC-N, Rib-N, and Alp1-N) boosted by the second dose (Figure 2A). This lack of boost effect is thus contrasting the heightened IgG response observed after the second dose. Also in contrast to IgG, all Alp-Ns displayed equally strong correlation between pre- and post-vaccination IgA levels, with Pearson correlation coefficients in the same range as those achieved for IgG against Alp1-N and Alp2/3-N (Figure 2B). Consistent with this, the difference in fold increase between the homotypic and heterotypic Alp-Ns was not equally pronounced for IgA as observed for IgG; the fold increase in IgA levels was in the lower range for all individual Alp-Ns (Table S1). This indicates that all IgA responses to the vaccine depend on homologous pre-existing IgA immunity, present in some but not all subjects.

GBS-NN does not induce substantial IgM production

Because IgM can efficiently enhance OPk and is not transferred across the placenta, it was important to measure IgM for adequate interpretation of OPkA results (see below). Here we only assessed responses against the homotypic Alp-Ns. Relatively high levels of Alp-N-specific IgM were present in pre-vaccination sera (Table S1). Although vaccination resulted in a significantly increased GMC of IgM against Rib-N (GMC 1.16 versus 1.42 μg/mL for day 0 and day 57, respectively; $p = 0.0022$) and αC-N (GMC 0.89 versus 1.65 μg/mL for day 0 and day 57, respectively; $p < 0.0001$), this represented only a 1.2-fold and 1.9-fold increase when comparing post- and pre-vaccination levels against Rib-N and αC-N, respectively (Table S1).

Vaccine-induced Ab levels correlate with binding to native αC and Rib proteins on bacteria

To assess whether Ab concentrations against the recombinant Alp-Ns reflect binding to native αC and Rib proteins on bacteria, we used flow cytometry to measure bacterial IgG binding after incubation with sera from subjects selected to represent the whole range of post-vaccination IgG concentrations. We first verified a wide linear range of the assay (data not shown) and then assayed all sera at a fixed dilution (1/240). Because most pre-vaccination sera contain IgG against GBS antigens distinct from αC-N and Rib-N, we subtracted the geometric mean fluorescent intensity (GMFI) obtained for the pre-vaccination serum from the GMFI obtained for the corresponding post-vaccination serum. The resulting ΔGMFI represents the vaccine-induced increase in IgG binding to the bacteria. We applied the same procedure to the IgG concentrations measured by ELISA to obtain the vaccine-induced IgG concentration (ΔIgG concentration). Vaccine-induced αC-N- and Rib-N-specific IgG levels correlated strongly with the ΔGMFI obtained for A909 and BM110, respectively (Figures 3A and 3B). The results confirm that detection of Alp-N-specific Abs

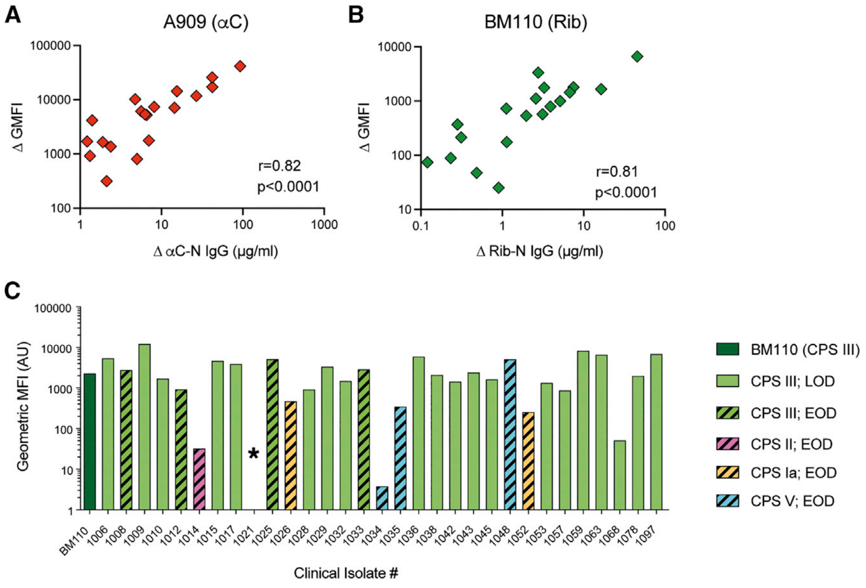

Figure 3. Vaccine-induced Alp-N-specific IgG correlates with an increase in IgG binding to intact bacteria and detects Rib protein on the surface of clinical isolates with different CPS types

(A and B) Binding of human serum IgG to strain A909 (A) and BM110 (B) was analyzed by flow cytometry before and after vaccination, and the association between the vaccine-induced increase in GMFI (ΔGMFI) and vaccine-induced increase in corresponding IgG concentration was assessed by Pearson correlation.

(C) Flow cytometry analysis of Rib protein on the surface of 31 clinical EOD and LOD isolates possessing the Rib gene and indicated CPS types, using a fixed dilution (1:3,200) of a rabbit GBS antiserum. The model strain BM110 was included as reference. Results show geometric mean fluorescence intensities (GMFIs). The asterisk for strain 1,021 indicates bimodal expression (one negative and one positive GBS population; it is not possible to report GMFI).

in ELISA, using recombinant Alp-N proteins, reflects binding to native Alps on the bacterial surface.

Next we assessed whether GBS-NN specific Abs were able to bind to a panel of 31 invasive GBS isolates possessing the gene for Rib and collected from EOD and LOD cases in Johannesburg during 2012–2014.[34] The presence of Rib and absence of other Alp genes was confirmed by PCR (data not shown). The CPS types had been determined previously and included type Ia, II, III, and V.[34] To avoid artificial results because of pre-existing Abs in sera from human vaccinees, we used a rabbit GBS-NN antiserum.[11] Using a fixed dilution at 1:3,200 of the antiserum (optimal dilution in relation to the linear range of the assay), flow cytometry analysis detected Rib protein on all isolates, albeit at varying levels (Figure 3C). Abs elicited by the vaccine therefore bind to Rib on clinical GBS isolates of different CPS types and collected from EOD and LOD cases.

IgG responses elicited by GBS-NN mediate OPk of GBS

We assessed the ability of pre-versus post-vaccination sera from all study participants to mediate bacterial killing in an OPkA.[35] Target bacterial strains were selected to cover all individual Alp-Ns and included the strains A909 (αC; CPS serotype Ia), BM110 (Rib; CPS serotype III), NCTC12906 (Alp1; CPS serotype Ia), and NEM316 (Alp2; CPS serotype III). Because the N-domain of Alp3 is identical to that of Alp2, a separate Alp3-expressing strain was not included. As shown in Figure 4A, most pre-vaccination sera mediated killing that was directly quantifiable within the serum dilution range analyzed; 77% of the subjects had an OPkA titer of 10 or greater against the αC-expressing strain A909 and 83%, 79%, and 90% of the pre-vaccination sera displayed such quantifiable titers against the strains expressing Rib, Alp1, and Alp2, respectively. Of note, the isotype and specificities of the opsonophagocytic Abs in these pre-immune sera are not known but are likely to include IgM, which cannot cross the placenta, and other Abs against the capsule and multiple protein GBS antigens.

Analysis of sera from the same subjects 4 weeks after the second dose revealed a significantly increased OPkA geometric mean titer (GMT) against all target strains (Figure 4A; Table 1). The GMTs increased from baseline values of 35 and 87 against A909 and BM110, respectively, to post-vaccination GMTs of 599 and 236 against the respective strain. This corresponds to a 17- and 2.7-fold increase in GMT against A909 and BM110, respectively. The vaccine-induced increase in OPk also occurred among subjects with relatively poor opsonophagocytic activity in their pre-vaccination sera, reflected by a higher proportion of the subjects reaching the arbitrarily set threshold titers of 50 and 100, respectively, after vaccination (Figure 4B). For the heterotypic strain NCTC12906 (Alp1), the GMT increased from a value of 61 before vaccination to 121 after vaccination, corresponding to a 2-fold increase in GMT. The GMT against NEM316 (Alp2) was 113 prior to vaccination and increased to 199 after vaccination, which corresponds to only a 1.8-fold increase in GMT. For these heterotypic strains, the proportion of subjects reaching the threshold titers of 50 and 100 was increased only marginally (NCTC12906; Alp1) or not at all (NEM316; Alp2) following vaccination (Figure 4B).

To assess the association between Alp-N-specific Ab levels and increased OPk induced by the vaccine, we calculated the vaccine-induced OPkA titer (ΔOPkA titer) for each subject by subtracting the pre-from the post-vaccination titer. This allowed us to distinguish OPk activity induced by the vaccine from the activity already present prior to vaccination. We confirmed this approach by pre-incubating post-vaccination sera with excess soluble GBS-NN protein before conducting the OPkA, which reduced the post-vaccination OPk to the level observed for the corresponding pre-vaccination serum (Figures 4C and 4D; Table 2). This shows that vaccine-specific Abs accounted for the increased OPkA titers observed after vaccination. Of note, for some subjects, analogous adsorption of the pre-vaccination serum resulted in partial inhibition of the pre-existing opsonophagocytic serum activity (Table 2), indicating that naturally

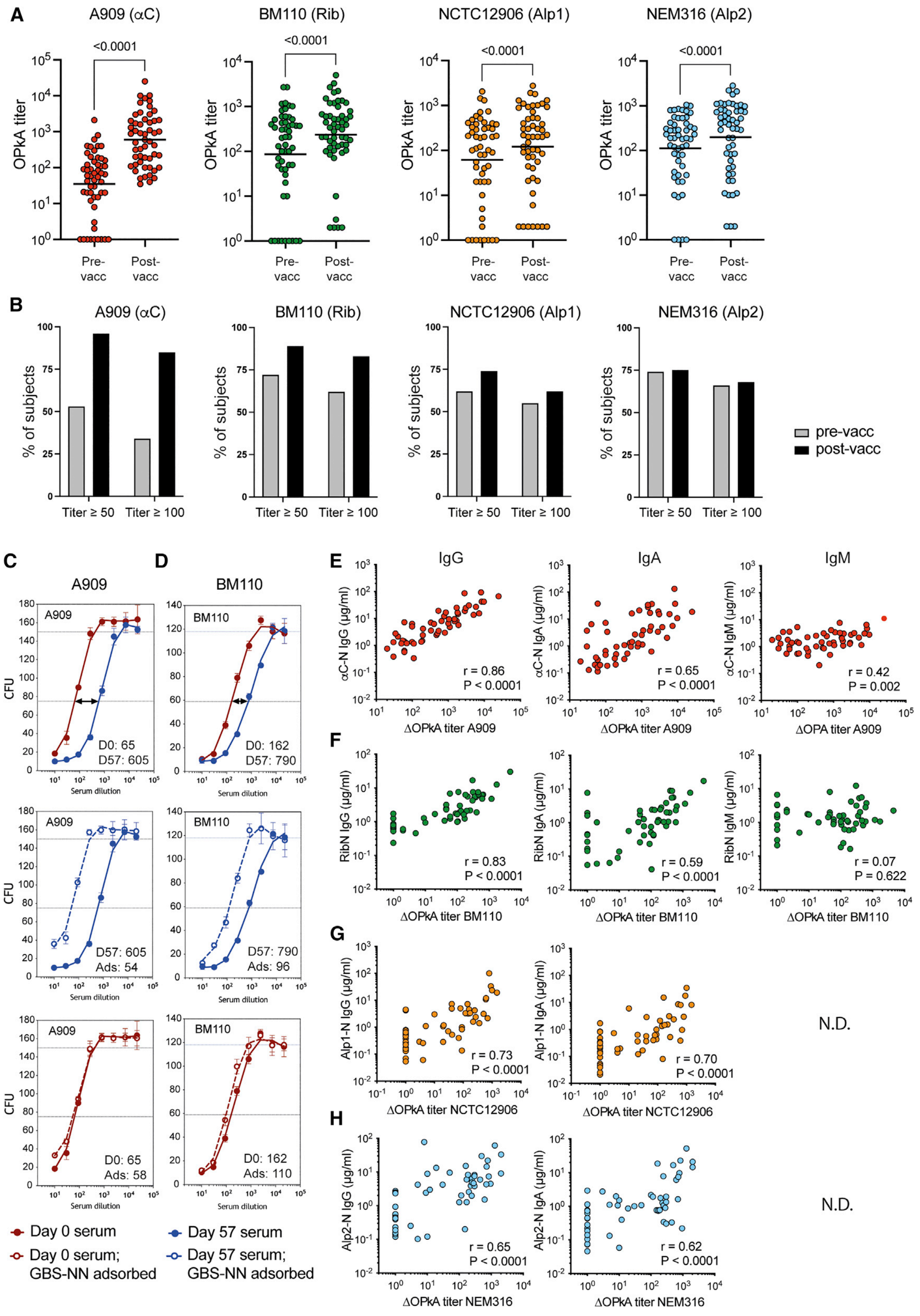

(legend on next page)

CellPress
OPEN ACCESS

Table 1. OPk of GBS strains with expression of the indicated Alp family member

Strain name	Alp expression	OPk GMT[a] (95% CI) Day 0	Day 57	Fold increase (95% CI)
A909	αC	**35** *(20–62)*	**599** *(376–955)*	**17.2** *(11.0–26.9)*
BM110	Rib	**87** *(45–168)*	**236** *(139–398)*	**2.7** *(1.9–3.9)*
NCTC12906	Alp1	**61** *(33–115)*	**121** *(68–217)*	**2.0** *(1.5–2.5)*
NEM316	Alp2	**113** *(68–188)*	**199** *(117–337)*	**1.8** *(1.5–2.1)*

Serum samples are from same cohorts and subjects as shown in Figures 1 and 2. GMTs and fold increase are shown in bold, with 95% CI in italics.
[a]Geometric mean OPkA titer (reciprocal serum dilution yielding 50% killing).

acquired Abs against αC-N and Rib-N can contribute to the killing detected prior to vaccination.

For the homotypic strains A909 and BM110, ΔOPkA titers correlated strongly with the IgG response against αC-N and Rib-N, respectively (Figures 4E and 4F). Moderate correlations were also detected between the ΔOPkA titer and the homologous IgA response (Figures 4E and 4F), probably because of a rather strong association between IgA and IgG responses ($r = 0.73$, $p > 0.0001$ for αC-N and $r = 0.65$, $p < 0.0001$ for Rib-N; data not shown). The ΔOPkA titers, however, correlated only weakly (A909) or not at all (BM110) with homologous Alp-N post-vaccination IgM levels (Figures 4E and 4F), in line with the poor IgM response induced by the vaccine. Similar results were obtained for the heterotypic strains NCTC12906 (Alp1) and NEM316 (Alp2), where ΔOPkA titers correlated with IgG and IgA directed against the Alp-N domain expressed by the respective strain (Figures 4G and 4H). IgM responses against these domains were not assessed. Finally, we assessed whether invasive GBS isolates, possessing the αC gene in combination with CPS type Ia, Ib, II, or V, were equally sensitive to killing as the strain A909. Here we selected two subjects with high levels of post-vaccination IgG against αC-N and, for each subject, determined the ΔOPkA titers against four invasive EOD isolates obtained from the abovementioned South African cohort.[34] Vaccine-induced Abs from both subjects mediated killing of all isolates to at least the same extent as observed for A909 and irrespective of capsule type (Table S2). The results indicate that the IgG response elicited by GBS-NN mediates efficient OPk of GBS strains expressing vaccine homotypic Alps, with some, but more variable, effect on the Alp heterotypic strains.

Abs elicited by GBS-NN prevent GBS invasion of cervical epithelial cells

To determine whether sera from vaccinated subjects prevent GBS invasion of cervical epithelial ME180 cells, in initial experiments we assessed four subjects with intermediate to high levels of post-vaccination Abs against αC-N and Rib-N (Table S3). For each subject, post-vaccination serum diluted 1/100 completely prevented invasion by the A909 and BM110 strains into ME180 cells (Figure 5A). The corresponding pre-vaccination serum did not have this effect or reduced invasion only partially. To assess whether the ability to interfere with bacterial invasion is generic for Abs induced by the GBS-NN vaccine, we designed experiments to minimize the effect of varying Ab concentrations among the vaccinees. First, we assessed the concentration of Alp-N specific Abs (the sum of IgG and IgA) required to reach maximal inhibition and found that 50–100 ng/mL in the assay was, in general, sufficient (data not shown). We then analyzed post-vaccination sera from a total of 28 randomly selected subjects after dilution of each serum to a target concentration of 100 ng/mL of specific IgG plus IgA. Corresponding pre-vaccination sera were diluted using the same dilution factor (irrespective of Ab concentration). In the presence of 100 ng/mL of vaccine-induced αC-N-specific IgG/IgA, A909 invasion was reduced to 3% relative to invasion occurring in the absence of serum (geometric mean value; 95% confidence interval [CI], 1.9%–4.5%) (Figure 5B). With the same dilution factor, the corresponding pre-vaccination sera reduced invasion to 60% relative to the no-serum control (95% CI, 44.3%–82.2%). For BM110, invasion was reduced to 6%, with post-vaccination sera diluted to 100 ng/mL of Rib-N-specific IgG/IgA (95% CI, 4.8–8.8%), whereas pre-vaccination sera diluted by the same factor again had a significantly less pronounced effect, lowering BM110 invasion to 74% relative to the no-serum control (95% CI, 55.1–98.9%) (Figure 5B). For both GBS strains and all subjects, post-vaccination serum inhibited invasion more efficiently compared with the corresponding serum collected prior to vaccination, except from one subject who already displayed a strong inhibitory effect against BM110 before administration of the vaccine. These results demonstrate that vaccination with GBS-NN broadly elicits

Figure 4. GBS-NN elicits an Ab response that mediates OPk of homotypic and heterotypic GBS strains
Pre- and post-vaccination sera were assessed for the ability to mediate OPk of the indicated GBS strains. OPkA titer is defined as the reciprocal serum dilution required to mediate 50% bacterial killing relative to killing in the absence of human serum.
(A) Total OPk with pre- and post-vaccination sera. Results show titers for individual sera and GMTs.
(B) Percentage of subjects reaching the indicated OPkA titer thresholds before and after vaccination.
(C and D) Representative OPk killing curves, showing colony-forming units (CFUs) for the A909 (C) and BM110 (D) strains after incubation with pre-vaccination (brown curves) or post-vaccination (blue curves) serum samples in the absence (filled circles) or presence (open circles) of 50 μg/ml soluble GBS-NN (adsorptions [Ads]). The top graphs show results for pre- versus post-vaccination sera in the absence of inhibitor. The center graphs show post-vaccination sera in the absence or presence of inhibitor. The bottom graphs show pre-vaccination sera in the absence or presence of inhibitor.
(E–H) Pearson correlations between ΔOPkA titer and concentrations of IgG (left), IgA (center), and IgM (right) specific for the strain homologous Alp-N.
See also Table S2.

Table 2. Inhibition of OPkA titers after pre-incubation of pre-immune (day 0) and post-vaccination (day 57) sera with soluble GBS-NN (50 μg/mL)

Subject ID	GBS target strain	Serum	AlpN-specific IgG (μg/mL)[a]	OPkA titer[b] No inhibition	OPkA titer[b] With inhibition	Inhibition (%)[c]	Inhibition of ΔOPkA (%)[d]
B30	BM110 (Rib)	preimmune	0.10	375	317	15	–
		**vaccinated**	**12.68**	**1,608**	**375**	**77**	**100**
B31	BM110 (Rib)	preimmune	0.06	35	9	74	–
		**vaccinated**	**6.51**	**1,108**	**10**	**99**	**102**
B09	BM110 (Rib)	preimmune	0.29	1	1	no titer	–
		**vaccinated**	**4.31**	**126**	**1**	**99**	**100**
B06	BM110 (Rib)	preimmune	0.11	125	106	15	–
		**vaccinated**	**16.97**	**518**	**131**	**75**	**98**
A02	A909 (αC)	preimmune	0.10	1	1	no titer	–
		**vaccinated**	**4.85**	**120**	**1**	**99**	**100**
B06	A909 (αC)	preimmune	0.22	146	190	0	–
		**vaccinated**	**14.64**	**2,216**	**214**	**90**	**97**
A03	A909 (αC)	preimmune	0.37	1	1	no titer	–
		**vaccinated**	**42.39**	**1,300**	**2**	**100**	**100**
A06	A909 (αC)	preimmune	0.16	264	217	18	–
		**vaccinated**	**42.03**	**5,692**	**149**	**97**	**102**

Results obtained with post-vaccination sera are shown in bold.
[a]Strain homologous to Alp-N (Rib-N or αC-N)-specific IgG serum concentration.
[b]Absolute OPkA titer achieved for the respective serum sample (reciprocal serum dilution yielding 50% killing).
[c]Reduction in OPkA titer in the presence of soluble GBS-NN (50 μg/mL) relative to the titer achieved for the same serum in the absence of inhibitor.
[d]Reduction in OPkA titer in the presence of soluble GBS-NN (50 μg/mL) relative to the vaccination-induced increase in OPkA titer (ΔOPkA titer = day 57 – day 0).

αC-N and Rib-N-specific Abs that possess the ability to inhibit GBS invasion of human cervical epithelial cells.

Placental transfer of naturally acquired Abs leads to accumulation of αC-N- and Rib-N-specific IgG in cord blood

Transfer of maternal Abs across the placenta relies on the neonatal Fc receptor, expressed by placental trophoblasts. Although restricted to IgG, the IgG1 subclass is transferred more efficiently than the other IgG subclasses.[33] Given the similar IgG1 dominance of naturally acquired and vaccine-induced Alp-N-specific IgG (Figure 1C), we conducted a separate study to assess the placental transfer rate of naturally acquired Alp-N-specific IgG as well as the longevity of these Abs in neonatal blood. To this end, we collected venous blood from pregnant women on the day of childbirth and paired cord blood samples (n = 152). In addition, paired maternal and neonatal blood samples were taken approximately 1 month (n = 104) and two months (n = 60) after birth. The GMC of αC-N-specific IgG in partum maternal sera was 93.7 ng/mL (Figure 6A). The corresponding value for cord blood was 113.8 ng/mL, a 122% enrichment of αC-N-specific IgG in the cord compared with maternal blood. The GMCs of Rib-N-specific IgG in the partum maternal sera and matched cord blood samples were 33.4 ng/mL and 37.4 ng/mL, respectively, corresponding to 112% enrichment in the cord blood samples (Figure 6B).

Alp-N-specific Ab levels decreased progressively in neonatal blood 1 and 2 months after birth (Figures 6A and 6B). Still, both specificities were detected at these later time points. Relative to cord blood, αC-N-specific IgG GMC dropped to 54% and 49% 1 and 2 months after birth, respectively. The corresponding values for Rib-N-specific IgG were 48% and 32%, respectively. Although Alp-N-specific IgG levels decreased gradually in neonatal blood, the opposite was true for postpartum maternal blood; 2 months after childbirth, the GMC of IgG against αC-N and Rib-N had increased to 191% and 187% of the levels detected at partum, respectively (Figures 6A and 6B). Using cord blood and neonatal blood samples collected ~1 month after delivery, we next determined the half-life of the Alp-N-specific IgG in neonatal blood. Taking the individual blood collection time points into account, the geometric mean half-life of αC-N and Rib-N-specific IgG in neonatal blood was calculated to be 40 and 38 days, respectively. These results demonstrate that naturally acquired maternal IgG against αC-N and Rib-N is transferred efficiently across the placenta, reaching a higher concentration in the cord compared with maternal blood, and persists for extended periods of time in neonatal blood.

DISCUSSION

We have recently reported on the immunogenicity of GBS-NN in a randomized, placebo-controlled, double-blind phase 1 trial in healthy adult women.[10] In the current study, we expanded our

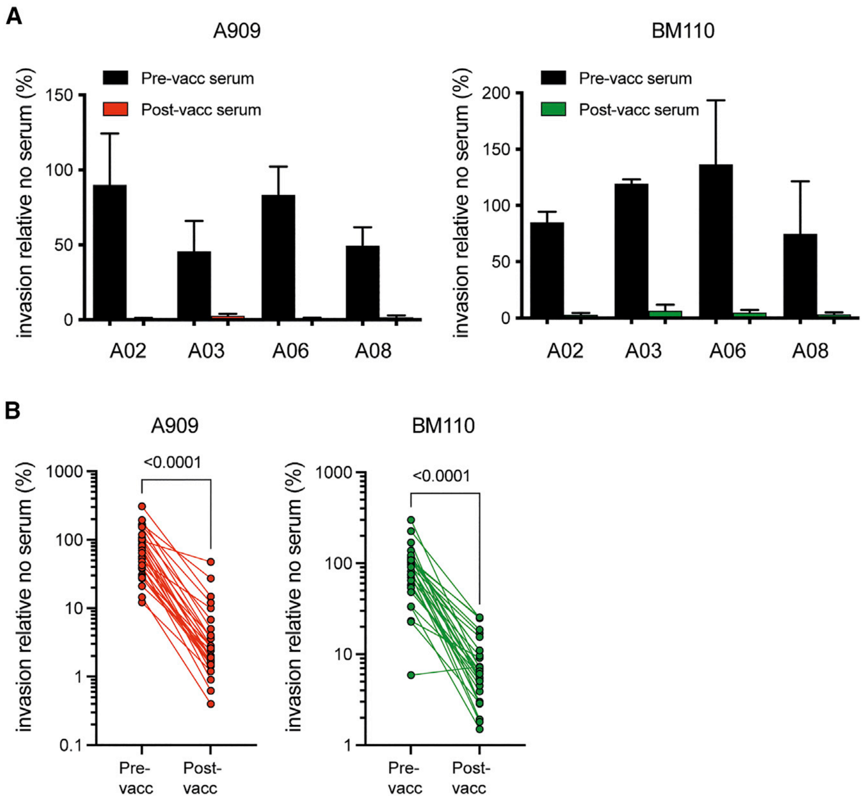

Figure 5. Post-vaccination sera prevent GBS invasion of cervical epithelial cells

(A) Post-vaccination sera from four subjects with intermediate to high levels of IgG and/or IgA against αC-N and Rib-N were compared with the paired pre-vaccination sera for the ability to prevent A909 (left) and BM110 (right) invasion of human cervical epithelial ME180 cells. All sera were diluted 1/100. Pooled results from three independent experiments. Error bars indicate SD. For information on αC-N- and Rib-N-specific Ab concentrations in selected sera, see Table S3.

(B) Post-vaccination sera were diluted to a target concentration of 100 ng/mL IgG plus IgA against αC-N (left) or Rib-N (right) and compared with corresponding pre-vaccination sera for the ability to prevent invasion of ME180 cells (n = 28). Each pre-vaccination serum was diluted by the same dilution factor as the paired post-vaccination serum. The geometric mean serum dilution factor for A909 experiments was 116 (range, 14–898). The geometric mean serum dilution factor for BM110 experiments was 38 (range, 11–204). All results are normalized to invasion in the absence of human serum.

characterization of the immune response elicited by the vaccine. We demonstrate that vaccination results in IgG and IgA responses against homotypic and heterotypic Alp-Ns, increased OPk activity, and an enhanced ability to prevent bacterial invasion of cervical epithelial cells. Using serum samples from a separate study cohort of paired non-vaccinated pregnant women and neonates, we show that naturally acquired IgG against αC-N and Rib-N reaches a higher concentration in cord relative to partum maternal blood and persists in neonatal blood with an extended half-life of ∼40 days.

Subjects with relatively low levels of pre-existing Abs against the homotypic Alp-Ns achieved high levels of IgG against these domains after two doses of the vaccine. We have shown recently that the robust response against GBS-NN in subjects with low pre-existing IgG levels is related to a highly beneficial effect of a second dose in these subjects.[10] Subjects with high levels of pre-existing IgG already reach a saturated IgG level after the first dose, indicating that the first dose induces an anamnestic response in these subjects. IgG responses against Alp1-N and Alp2/3-N displayed different characteristics because two doses induced relatively strong responses only in vaccinees with pronounced pre-existing levels of IgG against these heterotypic antigens. This suggests that GBS-NN reactivates heterotypic memory B cells in some subjects and that heterotypic responses are induced poorly in the absence of such memory B cells. Indeed, despite 60%–70% sequence homology between the different Alp-N domains,[16] animal experiments have demonstrated a lack of strong cross-reactivity between Alp-Ns.[12] Because memory B cells display a lower activation threshold than their naive counterparts,[36] it seems possible that low-affinity interactions with GBS-NN are sufficient to drive re-activation of heterotypic memory B cells but are inadequate for generation of heterotypic responses originating from the naive B cell compartment. An alternative (but not mutually exclusive) interpretation is that repeated colonization with strains expressing variant Alps, in some subjects, over time has selected for memory B cells with specificity for epitopes shared fully or partially by the different Alp-Ns. This phenomenon of repertoire freezing, or "original antigenic sin," was first described for consecutive responses against variant influenza virus strains and subsequently also for other viral infections, including HIV and dengue virus.[37,38] Nevertheless, our results show that a vaccine based on only αC-N and Rib-N will not stimulate sufficiently strong responses against Alp1-N and Alp2/3-N in subjects lacking pre-existing reactivity against these domains. We have therefore now included a second Alp1-N-Alp2/3-N fusion protein in the vaccine formulation, and a phase I study of this second-generation vaccine was recently completed. Results from this study will be reported elsewhere.

Several studies have assessed the distribution of individual Alps among colonizing GBS isolates. Although frequencies vary somewhat globally, Rib and αC appear to be the most common variants.[12] The high concentrations of naturally acquired Abs against Alp2/3-N detected in the current study was therefore surprising. It is, however, unclear which antigens underlie the pre-existing Alp2/3-N serum reactivity. Alp3 is essentially identical to the R28 protein expressed by group A *Streptococcus* (GAS) of the *emm28* genotype,[16,27] one of the most prevalent invasive GAS genotypes in Europe.[39–41] R28 is also present in some clinical isolates of *Streptococcus dysgalactiae* subspecies

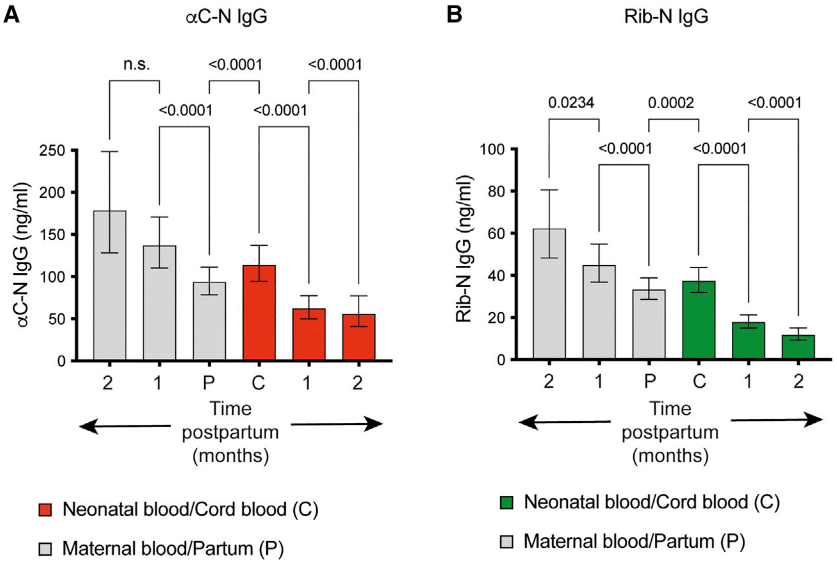

Figure 6. Naturally acquired IgG against αC-N and Rib-N accumulates in neonatal blood and persists for at least 2 months

(A and B) Serum concentrations of IgG against αC-N (A) and Rib-N (B) in a cohort of paired mothers and neonates. Maternal venous blood and cord blood were collected at partum (n = 152), as well as approximately 1 month (n = 105), and approximately two months (n = 61) after delivery, respectively (STAR Methods). Results shown are IgG GMC ±95% CI. Statistical analysis was performed by paired t test on logarithmically transformed concentration values. For each comparison, only paired values were included in the analysis (maternal partum versus cord blood, n = 152; partum/cord versus 1 month, n = 105; 1 month versus two months, n = 61).

equisimilis (SDSE),[42] a streptococcal species with steadily increasing prevalence, causing infections similar to those associated with GAS.[43] It seems possible that carriage of and/or infection with R28-expressing strains of GAS or SDSE could have contributed to the high levels of naturally acquired Alp2/3-N-reactive Abs detected in the current study.

Intramuscular immunization with GBS-NN induced significant but varying levels of IgA against all four Alp-Ns. Like the vaccine heterotypic IgG response, increased concentrations of Alp-N-specific IgA were observed only in vaccinees with pre-existing IgA against the homologous Alp-N. The correlation with pre-existing immunity was strong for IgA responses against all four Alp-Ns, in marked contrast to the vaccine-induced IgG levels where only responses against the heterotypic domains displayed similarly strong correlations. IgA responses develop mostly in mucosa-associated lymphoid tissues,[44] and it seems possible that that the strong association between vaccine-induced and pre-existing IgA reflects reactivation of IgA^{+} memory B cells originating from mucosal tissues. The functional relevance of the IgA response elicited by parenteral immunization with GBS-NN remains to be determined.

Post-vaccination sera displayed a pronounced increase in OPk activity against GBS strains expressing vaccine homotypic or heterotypic Alps. Opsonophagocytic Ab titers have been assessed to evaluate the efficacy of pneumococcal vaccines and appear to represent a more stringent serocorrelate of protection against pneumococcal disease than Ab binding titers.[32] Indeed, accelerated licensure of the higher-valency Prevnar 13 conjugate vaccine for use in adults older than 50 years was approved based on OPkA titers as an immunological surrogate endpoint (Centers for Disease Control and Prevention [CDC], the Pink Book, Pneumococcal Disease; https://www.cdc.gov/vaccines/pubs/pinkbook/pneumo.html). As a phase III efficacy study of a maternal GBS vaccine candidate would require a large study cohort accelerated licensure and vaccine approval based on OPkA titers have also been discussed in this case.[30] Difficulty demonstrating efficient OPk activity of Abs against protein antigens has been a concern in the pneumococcal field and has hampered development of a capsular serotype-independent, protein-based pneumococcal vaccine.[45–47]

The present study demonstrates that the Alps of GBS are relevant targets for opsonophagocytic Ab responses and that these responses are readily quantifiable in the OPkA. Although based on a limited number of isolates, our results show that GBS-NN-specific Abs bind to a panel of clinical isolates possessing the Rib gene in combination with different CPS types and that vaccine-induced Abs mediate efficient killing of clinical EOD isolates possessing the αC gene irrespective of capsule type. Although protective OPkA titer thresholds have not yet been established for neonatal GBS disease, there are currently international efforts to generate a standardized OPkA for GBS.[30] Such an assay should facilitate the process of defining a protective OPkA titer threshold and should also allow direct comparisons of serum OPk activity conferred by protein- versus CPS-based vaccine formulations.

Although future phase II and III studies should assess OPkA titers in cord blood to reflect activity transferred to the neonate, the increased killing we observed for post-vaccination sera correlated strongly with the homologous Alp-N-specific IgG response but not with corresponding IgM levels. Furthermore, IgG responses were completely dominated by IgG1, representing the IgG subclass that is transferred most efficiently across the placenta.[33] These findings indicate that the opsonophagocytic Abs induced by GBS-NN will be transferred efficiently to the infant. This notion is supported by our demonstration that naturally acquired IgG against αC-N and Rib-N, which were also of the IgG1 subclass, reached higher concentrations in cord relative to partum maternal blood. Similarly high transfer rates have been reported previously for IgG against other protein antigens but not for anti-CPS Abs induced by investigational GBS glycoconjugate vaccines.[48,49] Of note, the maternal serum concentration of naturally acquired αC-N- and Rib-N-specific IgG increased 1–2 months after childbirth, probably reflecting gradual normalization of the plasma volume, which is highly expanded across gestation.[50] Although neonatal serum levels declined instead during this period, IgG of both Alp-N specificities was still detected in the neonatal blood 2 months after birth

and displayed an extended half-life of ∼40 days, in line with previous reports on neonatal serum IgG half-life.[49,51]

Abs elicited by the vaccine prevented GBS internalization into cervical epithelial cells, and this was evident for all subjects assessed. Although several studies have demonstrated invasion through internalization of GBS into host cells, a paracellular pathway has also been described,[28] and it has been shown recently that GBS induces epithelial exfoliation to sustain colonization and increase bacterial dissemination in a murine model of ascending infection.[52] Similar to the intracellular invasion pathway,[26] exfoliation was dependent on $\alpha_1\beta_1$ integrin, but the GBS-associated molecules interacting with the integrin were not identified.[52] The relative contribution of exfoliation versus direct invasion of the epithelium during ascending infections, as well as the possible involvement of the Alps in triggering $\alpha_1\beta_1$ integrin-dependent exfoliation, merit further investigations.

We did not investigate invasion by GBS strains expressing Alp1-3. However, as mentioned above, the N-terminal domains of Alp2/3 and the R28 protein of GAS are identical, and the latter mediates GAS adhesion to cervical, endometrial, and pulmonary epithelial cells as well as to endometrial stromal cells through interactions with the $\alpha_3\beta_1$, $\alpha_6\beta_1$, and $\alpha_6\beta_4$ integrins.[27,53] In addition, R28 was common among GAS strains isolated from recent outbreaks of puerperal fever in Great Britain,[41] which has led to the hypothesis that the protein confers tissue tropism for the female genital tract.[54] It therefore seems likely that all Alp family members possess an ability to mediate adherence to or invasion of host cells. Because GBS has been demonstrated to invade not only epithelial cells of the female genital tract but also neonatal respiratory and lung epithelial cells as well as brain microvascular endothelial cells, it will be important to investigate each individual Alp family member in relation to the ability of different GBS strains to cross these epithelial and endothelial barriers.

We show that vaccination with GBS-NN elicits Abs that target GBS strains with homotypic and heterotypic Alp expression but that the heterotypic responses rely on pre-existing immunity. The Abs induced through vaccination mediate OPk that is quantifiable in the OPkA, inhibit bacterial invasion of human cervical epithelial cells, and are likely to be transferred efficiently across the placenta. The results reveal a requirement to include Alp1-N and Alp2/3-N in the vaccine formulation to improve responses against these Alps in subjects with low pre-existing immunity. This should ensure adequate responses to all individual Alp serotypes and, hence, broaden the vaccine coverage to close to 100% of clinical GBS isolates.

Limitations of the study

Although we show that the GBS-NN vaccine elicits strong and opsonophagocytic Ab responses against αC-N and Rib-N in healthy adult non-pregnant women, protective thresholds in relation to neonatal disease remain to be established. Such thresholds should preferentially be established for neonatal blood (and/or cord blood) to reflect the levels and functionality of Abs being transferred to neonates. Furthermore, the current vaccine formulation does not include the N-terminal domains of Alp1 and Alp2/3. Although our results demonstrate that GBS-NN also elicits functional Abs against these domains in subjects with pre-existing immunity, inclusion of Alp1-N and Alp2/3-N in the vaccine formulation will be required to support robust responses in all subjects.

STAR★METHODS

Detailed methods are provided in the online version of this paper and include the following:

- KEY RESOURCES TABLE
- RESOURCE AVAILABILITY
  - Lead contact
  - Materials availability
  - Data and code availability
- EXPERIMENTAL MODEL AND SUBJECT DETAILS
  - Human subjects
  - Bacterial strains
  - Cell lines
- METHOD DETAILS
  - Cloning and production of recombinant Alp-N proteins
  - Purification of recombinant Alp-N proteins
  - *Quantitation of Alp-N-specific* IgG, IgG1, IgG2 and IgA *in sera from the study subjects*
  - Quantitation of Alp-N specific IgM in sera from the study subjects
  - Opsonophagocytic killing assay (OPkA)
  - Inhibition of serum OPkA activity by GBS-NN
  - Assessment of binding of Alp-N-specific serum IgG to whole GBS by flow cytometry (FACS)
  - Epithelial cell invasion inhibition assay
- QUANTIFICATION AND STATISTICAL ANALYSIS
- ADDITIONAL RESOURCES

SUPPLEMENTAL INFORMATION

Supplemental information can be found online at https://doi.org/10.1016/j.xcrm.2022.100511.

ACKNOWLEDGMENTS

The authors wish to thank Nina Olsen Nathan for assistance with writing the study protocol and application for the ethics permit for the study of maternal/neonatal naturally acquired Abs. The authors also wish to thank Hilal Vural, Gitte Gulloev, Charlotte Draeger, and Bettina Pia Malling (Department of Obstetrics, Copenhagen University Hospital, Rigshospitalet, Copenhagen, Denmark), as well as the staff at the maternity ward and postnatal ward for invaluable help with sample collection and preparation. We thank the Protein Expression Group (Bioneer A/S, Denmark) for cloning, expression, and purification of the Alp-N antigens used in this study. We also wish to thank Shabir Madhi and Gaurav Kwatra (University of the Witwatersrand, South Africa) for kindly providing clinical GBS isolates obtained from cases of neonatal invasive disease. Finally, we thank Sharné van Dijl for help with the graphical abstract. This study was supported by the European Commission Seventh Framework Program (NeoStrep, grant agreement 601743).

AUTHOR CONTRIBUTIONS

A.P., P.B.F., G.K., M.D., and B.J.-L. conceived the study. A.P., J.L., M.G.-M., D.C., and S.L. performed the experiments. A.P., J.L., M.D., and B.J.-L. analyzed data. A.L.R. and M.H. collected, organized, and provided critical biological samples. B.J.-L., J.J.P., and P.B.F. supervised the project. P.B.F. and B.J.-L. secured funding. B.J.-L. and A.P. wrote the manuscript. All authors reviewed and edited the manuscript.

DECLARATION OF INTERESTS

B.J.-L. reports personal fees and grants from MinervaX ApS during the conduct of the study. P.B.F. reports grants and personal fees from MinervaX ApS during the conduct of the study and personal fees from MinervaX ApS outside of the submitted work. In addition, P.B.F. has a pending patent application relating to use of GBS-NN as a vaccine candidate. M.D. reports personal fees from MinervaX ApS during the conduct of the study. G.K. reports personal fees from MinervaX ApS during the conduct of the study and personal fees from various clients outside of the submitted work.

Received: September 9, 2021
Revised: November 17, 2021
Accepted: January 7, 2022
Published: February 15, 2022

REFERENCES

1. Kwatra, G., Cunnington, M.C., Merrall, E., Adrian, P.V., Ip, M., Klugman, K.P., Tam, W.H., and Madhi, S.A. (2016). Prevalence of maternal colonisation with group B streptococcus: a systematic review and meta-analysis. Lancet Infect. Dis. *16*, 1076–1084. https://doi.org/10.1016/S1473-3099(16)30055-X.
2. Russell, N.J., Seale, A.C., O'Driscoll, M., O'Sullivan, C., Bianchi-Jassir, F., Gonzalez-Guarin, J., Lawn, J.E., Baker, C.J., Bartlett, L., Cutland, C., et al. (2017). Maternal colonization with group b streptococcus and serotype distribution worldwide: systematic review and meta-analyses. Clin. Infect. Dis. *65*, S100–S111. https://doi.org/10.1093/cid/cix658.
3. Seale, A.C., Bianchi-Jassir, F., Russell, N.J., Kohli-Lynch, M., Tann, C.J., Hall, J., Madrid, L., Blencowe, H., Cousens, S., Baker, C.J., et al. (2017). Estimates of the burden of group b streptococcal disease worldwide for pregnant women, stillbirths, and children. Clin. Infect. Dis. *65*, S200–S219. https://doi.org/10.1093/cid/cix664.
4. Seale, A.C., Blencowe, H., Bianchi-Jassir, F., Embleton, N., Bassat, Q., Ordi, J., Menéndez, C., Cutland, C., Briner, C., Berkley, J.A., et al. (2017). Stillbirth with group b streptococcus disease worldwide: systematic review and meta-analyses. Clin. Infect. Dis. *65*, S125–S132. https://doi.org/10.1093/cid/cix585.
5. Hall, J., Adams, N.H., Bartlett, L., Seale, A.C., Lamagni, T., Bianchi-Jassir, F., Lawn, J.E., Baker, C.J., Cutland, C., Heath, P.T., et al. (2017). Maternal disease with group b streptococcus and serotype distribution worldwide: systematic review and meta-analyses. Clin. Infect. Dis. *65*, S112–S124. https://doi.org/10.1093/cid/cix660.
6. Bianchi-Jassir, F., Seale, A.C., Kohli-Lynch, M., Lawn, J.E., Baker, C.J., Bartlett, L., Cutland, C., Gravett, M.G., Heath, P.T., Ip, M., et al. (2017). Preterm birth associated with group B streptococcus maternal colonization worldwide: systematic review and meta-analyses. Clin. Infect. Dis. *65*, S133–S142. https://doi.org/10.1093/cid/cix661.
7. Verani, J.R., McGee, L., and Schrag, S.J.Division of Bacterial Diseases; Centers for Disease Control and Prevention (CDC) (2010). Prevention of perinatal group B streptococcal disease–revised guidelines from CDC, 2010. MMWR Recomm Rep. 59, 1–36.
8. Berardi, A., Rossi, C., Lugli, L., Creti, R., Bacchi Reggiani, M.L., Lanari, M., Memo, L., Pedna, M.F., Venturelli, C., Perrone, E., et al. (2013). Group B streptococcus late-onset disease: 2003-2010. Pediatrics *131*, e361–e368. https://doi.org/10.1542/peds.2012-1231.
9. Seale, A.C., Baker, C.J., Berkley, J.A., Madhi, S.A., Ordi, J., Saha, S.K., Schrag, S.J., Sobanjo-Ter Meulen, A., and Vekemans, J. (2019). Vaccines for maternal immunization against Group B Streptococcus disease: WHO perspectives on case ascertainment and case definitions. Vaccine *37*, 4877–4885. https://doi.org/10.1016/j.vaccine.2019.07.012.
10. Fischer, P., Pawlowski, A., Cao, D., Bell, D., Kitson, G., Darsley, M., and Johansson-Lindbom, B. (2021). Safety and immunogenicity of a prototype recombinant alpha-like protein subunit vaccine (GBS-NN) against Group B Streptococcus in a randomised placebo-controlled double-blind phase 1 trial in healthy adult women. Vaccine *39*, 4489–4499. https://doi.org/10.1016/j.vaccine.2021.06.046.
11. Stålhammar-Carlemalm, M., Waldemarsson, J., Johnsson, E., Areschoug, T., and Lindahl, G. (2007). Nonimmunodominant regions are effective as building blocks in a streptococcal fusion protein vaccine. Cell Host Microbe *2*, 427–434. https://doi.org/10.1016/j.chom.2007.10.003.
12. Maeland, J.A., Afset, J.E., Lyng, R.V., and Radtke, A. (2015). Survey of immunological features of the alpha-like proteins of Streptococcus agalactiae. Clin. Vaccin. Immunol. *22*, 153–159. https://doi.org/10.1128/CVI.00643-14.
13. McGee, L., Chochua, S., Li, Z., Mathis, S., Rivers, J., Metcalf, B., Ryan, A., Alden, N., Farley, M.M., Harrison, L.H., et al. (2021). Multistate, population-based distributions of candidate vaccine targets, clonal complexes, and resistance features of invasive group B streptococci within the United States, 2015–2017. Clin. Infect. Dis. *72*, 1004–1013. https://doi.org/10.1093/cid/ciaa151.
14. Larsson, C., Stålhammar-Carlemalm, M., and Lindahl, G. (1996). Experimental vaccination against group B streptococcus, an encapsulated bacterium, with highly purified preparations of cell surface proteins Rib and alpha. Infect. Immun. *64*, 3518–3523. https://doi.org/10.1128/iai.64.9.3518-3523.1996.
15. Larsson, C., Stålhammar-Carlemalm, M., and Lindahl, G. (1999). Protection against experimental infection with group B streptococcus by immunization with a bivalent protein vaccine. Vaccine *17*, 454–458. https://doi.org/10.1016/s0264-410x(98)00218-7.
16. Lachenauer, C.S., Creti, R., Michel, J.L., and Madoff, L.C. (2000). Mosaicism in the alpha-like protein genes of group B streptococci. Proc. Natl. Acad. Sci. U S A *97*, 9630–9635. https://doi.org/10.1073/pnas.97.17.9630.
17. Rubens, C.E., Raff, H.V., Jackson, J.C., Chi, E.Y., Bielitzki, J.T., and Hillier, S.L. (1991). Pathophysiology and histopathology of group B streptococcal sepsis in Macaca nemestrina primates induced after intraamniotic inoculation: evidence for bacterial cellular invasion. J. Infect. Dis. *164*, 320–330. https://doi.org/10.1093/infdis/164.2.320.
18. Moyo, S.R., Hägerstrand, I., Nyström, L., Tswana, S.A., Blomberg, J., Bergström, S., and Ljungh, A. (1996). Stillbirths and intrauterine infection, histologic chorioamnionitis and microbiological findings. Int. J. Gynaecol. Obstet. *54*, 115–123. https://doi.org/10.1016/0020-7292(96)02705-1.
19. Boggess, K.A., Watts, D.H., Hillier, S.L., Krohn, M.A., Benedetti, T.J., and Eschenbach, D.A. (1996). Bacteremia shortly after placental separation during cesarean delivery. Obstet. Gynecol. *87*, 779–784. https://doi.org/10.1016/0029-7844(96)00037-3.
20. Winram, S.B., Jonas, M., Chi, E., and Rubens, C.E. (1998). Characterization of group B streptococcal invasion of human chorion and amnion epithelial cells in vitro. Infect. Immun. *66*, 4932–4941. https://doi.org/10.1128/IAI.66.10.4932-4941.1998.
21. Nizet, V., Kim, K.S., Stins, M., Jonas, M., Chi, E.Y., Nguyen, D., and Rubens, C.E. (1997). Invasion of brain microvascular endothelial cells by group B streptococci. Infect. Immun. *65*, 5074–5081. https://doi.org/10.1128/iai.65.12.5074-5081.1997.
22. Rubens, C.E., Smith, S., Hulse, M., Chi, E.Y., and van Belle, G. (1992). Respiratory epithelial cell invasion by group B streptococci. Infect. Immun. *60*, 5157–5163. https://doi.org/10.1128/iai.60.12.5157-5163.1992.
23. Valentin-Weigand, P., Benkel, P., Rohde, M., and Chhatwal, G.S. (1996). Entry and intracellular survival of group B streptococci in J774 macrophages. Infect. Immun. *64*, 2467–2473. https://doi.org/10.1128/iai.64.7.2467-2473.1996.
24. Valentin-Weigand, P., Jungnitz, H., Zock, A., Rohde, M., and Chhatwal, G.S. (1997). Characterization of group B streptococcal invasion in HEp-2 epithelial cells. FEMS Microbiol. Lett. *147*, 69–74. https://doi.org/10.1111/j.1574-6968.1997.tb10222.x.
25. Bolduc, G.R., Baron, M.J., Gravekamp, C., Lachenauer, C.S., and Madoff, L.C. (2002). The alpha C protein mediates internalization of group B Streptococcus within human cervical epithelial cells. Cell Microbiol. *4*, 751–758. https://doi.org/10.1046/j.1462-5822.2002.00227.x.

26. Bolduc, G.R., and Madoff, L.C. (2007). The group B streptococcal alpha C protein binds alpha1beta1-integrin through a novel KTD motif that promotes internalization of GBS within human epithelial cells. Microbiology (Reading) *153*, 4039–4049. https://doi.org/10.1099/mic.0.2007/009134-0.

27. Stålhammar-Carlemalm, M., Areschoug, T., Larsson, C., and Lindahl, G. (1999). The R28 protein of Streptococcus pyogenes is related to several group B streptococcal surface proteins, confers protective immunity and promotes binding to human epithelial cells. Mol. Microbiol. *33*, 208–219. https://doi.org/10.1046/j.1365-2958.1999.01470.x.

28. Soriani, M., Santi, I., Taddei, A., Rappuoli, R., Grandi, G., and Telford, J.L. (2006). Group B Streptococcus crosses human epithelial cells by a paracellular route. J. Infect. Dis. *193*, 241–250. https://doi.org/10.1086/498982.

29. Baron, M.J., Bolduc, G.R., Goldberg, M.B., Aupérin, T.C., and Madoff, L.C. (2004). Alpha C protein of group B Streptococcus binds host cell surface glycosaminoglycan and enters cells by an actin-dependent mechanism. J. Biol. Chem. *279*, 24714–24723. https://doi.org/10.1074/jbc.M402164200.

30. Le Doare, K., Kampmann, B., Vekemans, J., Heath, P.T., Goldblatt, D., Nahm, M.H., Baker, C., Edwards, M.S., Kwatra, G., Andrews, N., et al. (2019). Serocorrelates of protection against infant group B streptococcus disease. Lancet Infect. Dis. *19*, e162–e171. https://doi.org/10.1016/S1473-3099(18)30659-5.

31. Larsson, C., Lindroth, M., Nordin, P., Stålhammar-Carlemalm, M., Lindahl, G., and Krantz, I. (2006). Association between low concentrations of antibodies to protein alpha and Rib and invasive neonatal group B streptococcal infection. Arch. Dis. Child Fetal Neonatal. Ed. *91*, F403–F408. https://doi.org/10.1136/adc.2005.090472.

32. Song, J.Y., Moseley, M.A., Burton, R.L., and Nahm, M.H. (2013). Pneumococcal vaccine and opsonic pneumococcal antibody. J. Infect. Chemother. *19*, 412–425. https://doi.org/10.1007/s10156-013-0601-1.

33. Malek, A., Sager, R., and Schneider, H. (1994). Maternal-fetal transport of immunoglobulin G and its subclasses during the third trimester of human pregnancy. Am. J. Reprod. Immunol. *32*, 8–14. https://doi.org/10.1111/j.1600-0897.1994.tb00873.x.

34. Dangor, Z., Lala, S.G., Cutland, C.L., Koen, A., Jose, L., Nakwa, F., Ramdin, T., Fredericks, J., Wadula, J., and Madhi, S.A. (2015). Burden of invasive group B Streptococcus disease and early neurological sequelae in South African infants. PLoS One *10*, e0123014. https://doi.org/10.1371/journal.pone.0123014.

35. Nahm, M.H., and Burton, R.L. (2016). Protocol for opsonophagocytic killing assay for antibodies against Group B *Streptococcus* (UAB GBS OPA).https://www.vaccine.uab.edu/uploads/mdocs/UAB-GBS-OPA.pdf.

36. Kurosaki, T., Kometani, K., and Ise, W. (2015). Memory B cells. Nat. Rev. Immunol. *15*, 149–159. https://doi.org/10.1038/nri3802.

37. Francis, T. (1960). On the doctrine of original antigenic sin. Proc. Am. Philos. Soc. *104*, 572–578.

38. Rothman, A.L. (2011). Immunity to dengue virus: a tale of original antigenic sin and tropical cytokine storms. Nat. Rev. Immunol. *11*, 532–543. https://doi.org/10.1038/nri3014.

39. Plainvert, C., Doloy, A., Loubinoux, J., Lepoutre, A., Collobert, G., Touak, G., Trieu-Cuot, P., Bouvet, A., Poyart, C., and network, C.-S. (2012). Invasive group A streptococcal infections in adults, France (2006-2010). Clin. Microbiol. Infect. *18*, 702–710. https://doi.org/10.1111/j.1469-0691.2011.03624.x.

40. Gherardi, G., Vitali, L.A., and Creti, R. (2018). Prevalent *emm* types among invasive GAS in Europe and North America since Year 2000. Front. Public Health *6*, 59. https://doi.org/10.3389/fpubh.2018.00059.

41. Colman, G., Tanna, A., Efstratiou, A., and Gaworzewska, E.T. (1993). The serotypes of Streptococcus pyogenes present in Britain during 1980-1990 and their association with disease. J. Med. Microbiol. *39*, 165–178. https://doi.org/10.1099/00222615-39-3-165.

42. Gherardi, G., Imperi, M., Palmieri, C., Magi, G., Facinelli, B., Baldassarri, L., Pataracchia, M., and Creti, R. (2014). Genetic diversity and virulence properties of Streptococcus dysgalactiae subsp. equisimilis from different sources. J. Med. Microbiol. *63*, 90–98. https://doi.org/10.1099/jmm.0.062109-0.

43. Brandt, C.M., and Spellerberg, B. (2009). Human infections due to Streptococcus dysgalactiae subspecies equisimilis. Clin. Infect. Dis. *49*, 766–772. https://doi.org/10.1086/605085.

44. Lycke, N.Y., and Bemark, M. (2017). The regulation of gut mucosal IgA B-cell responses: recent developments. Mucosal Immunol. *10*, 1361–1374. https://doi.org/10.1038/mi.2017.62.

45. Converso, T.R., Assoni, L., André, G.O., Darrieux, M., and Leite, L.C.C. (2020). The long search for a serotype independent pneumococcal vaccine. Expert Rev. Vaccin. *19*, 57–70. https://doi.org/10.1080/14760584.2020.1711055.

46. Ginsburg, A.S., Nahm, M.H., Khambaty, F.M., and Alderson, M.R. (2012). Issues and challenges in the development of pneumococcal protein vaccines. Expert Rev. Vaccin. *11*, 279–285. https://doi.org/10.1586/erv.12.5.

47. Daniels, C.C., Kim, K.H., Burton, R.L., Mirza, S., Walker, M., King, J., Hale, Y., Coan, P., Rhee, D.K., Nahm, M.H., and Briles, D.E. (2013). Modified opsonization, phagocytosis, and killing assays to measure potentially protective antibodies against pneumococcal surface protein A. Clin. Vaccin. Immunol *20*, 1549–1558. https://doi.org/10.1128/CVI.00371-13.

48. Calvert, A., and Jones, C.E. (2017). Placental transfer of antibody and its relationship to vaccination in pregnancy. Curr. Opin. Infect. Dis. *30*, 268–273. https://doi.org/10.1097/QCO.0000000000000372.

49. Madhi, S.A., Koen, A., Cutland, C.L., Jose, L., Govender, N., Wittke, F., Olugbosi, M., Sobanjo-Ter Meulen, A., Baker, S., Dull, P.M., et al. (2017). Antibody kinetics and response to routine vaccinations in infants born to women who received an investigational trivalent group B Streptococcus polysaccharide CRM197-conjugate vaccine during pregnancy. Clin. Infect. Dis. *65*, 1897–1904. https://doi.org/10.1093/cid/cix666.

50. Aguree, S., and Gernand, A.D. (2019). Plasma volume expansion across healthy pregnancy: a systematic review and meta-analysis of longitudinal studies. BMC Pregnancy Childbirth *19*, 508. https://doi.org/10.1186/s12884-019-2619-6.

51. Vilajeliu, A., Ferrer, L., Munrós, J., Goncé, A., López, M., Costa, J., Bayas, J.M., and Group, P.W. (2016). Pertussis vaccination during pregnancy: antibody persistence in infants. Vaccine *34*, 3719–3722. https://doi.org/10.1016/j.vaccine.2016.05.051.

52. Vornhagen, J., Armistead, B., Santana-Ufret, V., Gendrin, C., Merillat, S., Coleman, M., Quach, P., Boldenow, E., Alishetti, V., Leonhard-Melief, C., et al. (2018). Group B streptococcus exploits vaginal epithelial exfoliation for ascending infection. J. Clin. Invest. *128*, 1985–1999. https://doi.org/10.1172/JCI97043.

53. Weckel, A., Ahamada, D., Bellais, S., Méhats, C., Plainvert, C., Longo, M., Poyart, C., and Fouet, A. (2018). The N-terminal domain of the R28 protein promotes. J. Biol. Chem. *293*, 16006–16018. https://doi.org/10.1074/jbc.RA118.004134.

54. Areschoug, T., Carlsson, F., Stålhammar-Carlemalm, M., and Lindahl, G. (2004). Host-pathogen interactions in Streptococcus pyogenes infections, with special reference to puerperal fever and a comment on vaccine development. Vaccine *22*, S9–S14. https://doi.org/10.1016/j.vaccine.2004.08.010.

55. Creti, R., Fabretti, F., Orefici, G., and von Hunolstein, C. (2004). Multiplex PCR assay for direct identification of group B streptococcal alpha-protein-like protein genes. J. Clin. Microbiol. *42*, 1326–1329. https://doi.org/10.1128/JCM.42.3.1326-1329.2004.

STAR★METHODS

KEY RESOURCES TABLE

REAGENT or RESOURCE	SOURCE	IDENTIFIER
**Antibodies**		
Subcuvia 160mng/mL	Baxalta, Sweden	Lot: VNGN018A
Goat anti-human IgG (γ-chain) F(ab')2-HRP	Sigma-Aldrich	Cat#A2290
Goat anti-human IgA (α-chain)-HRP	Life Technologies	Cat#A18787
Goat anti-human IgM (μ-chain) F(ab')2-HRP	Sigma-Aldrich	Cat#A4290
Mouse anti-human IgG1 Fc-HRP	Life Technologies	Cat#MH1715
Mouse anti-human IgG2 Fd-HRP	Life Technologies	Cat#MH1722
Goat anti-human IgM (H+L) F(ab')2	Sigma-Aldrich	Cat#SAB3701393
Purified human serum IgM	InVitrogen	Cat#31146
goat anti-human IgM (μ-chain) F(ab')2-HRP	Sigma-Aldrich	Cat#A4290
FITC AffiniPure Donkey Anti-Human IgG (H+L)	Jackson ImmunoResearch	Cat#709-095-149
CD71 (Transferrin Receptor) Monoclonal Antibody (OKT9 (OKT-9)), PE,	eBioscience^{TM}	Cat#12-0719-42
CD35 Monoclonal Antibody (E11), APC	eBioscience^{TM}	Cat#17-0359-42
**Bacterial and virus strains**		
GBS strains A909 (AlpC/CPS type Ia)	Tomas Areschoug (Department of Laboratory Medicine, Lund University, Sweden)	N/A
GBS strains BM110 (Rib/CPS type III)	Tomas Areschoug (Department of Laboratory Medicine, Lund University, Sweden)	N/A
GBS strains NCTC12906 (Alp1/CPS type Ia)	Tomas Areschoug (Department of Laboratory Medicine, Lund University, Sweden)	N/A
GBS strains NEM316 (Alp2/ CPS type III)	Tomas Areschoug (Department of Laboratory Medicine, Lund University, Sweden)	N/A
E.coli BL21	Novagen	Cat#69450
**Biological samples**		
Baby rabbit complement (BRC; Pel-Freez, Arkansas)	Pel-freez Biologicals (Rogers, AR, USA)	Cat#31061
Clinical GBS isolates collected from neonatal disease cases	University of the Witwatersrand, Johannesburg, South Africa	Ref.[34]
**Chemicals, peptides, and recombinant proteins**		
AlphaC-N protein	Bioneer A/S, Denmark	Specific order/Batch number: 5422
Rib-N protein	Bioneer A/S, Denmark	Specific order/Batch number: 5474
Alp1-N protein	Bioneer A/S, Denmark	Specific order/Batch number: 5352
Alp2/3-N protein	Bioneer A/S, Denmark	Specific order/Batch number: 5400
Kanamycin	Sigma-Aldrich	Cat#K1377-25G
IPTG	Sigma-Aldrich	Cat#I6758
Tris-HCL	Sigma-Aldrich	Cat#PHG0002-5KG
Benzonase	Sigma-Aldrich	Cat#70746
PBS 10X	Medicago AB	Cat#12-9423-5
HiPrep^{TM} 26/10 Desalting	Sigma-Aldrich	Cat#GE17-5087-01
Tween 20	Merck	Cat#P2287

(*Continued on next page*)

Continued		
REAGENT or RESOURCE	SOURCE	IDENTIFIER
Sodio Acetate	Sigma-Aldrich	Cat# S2889-5KG
HCL	Sigma-Aldrich	Cat#320331-2.5L
NaCl	Sigma-Aldrich	Cat#S9888-10KG
3,3,5,5-tetramethylbenzidine (TMB) dihydrochloride hydrate	Sigma-Aldrich	Cat#T8768
H_2SO_4	Merck	Cat#258105
N, N-Dimethylformamide	Sigma-Aldrich	Cat#227056-100ML
Paraformaldehyde	Sigma-Aldrich	Cat#58127
Ethylenediaminetetraacetic acid (EDTA)	Sigma-Aldrich	Cat#E988
Bovine serum albumin (BSA)	Merck	Cat#A7906
Glycerol	Sigma-Aldrich	Cat#G7893-500ML
Trypsin-EDTA	Thermo Fisher scientific	Cat#25300054
Gentamycin	Sigma-Aldrich	Cat#G1397
Penicillin	Sigma-Aldrich	Cat#P3032
Triton X-100	Sigma-Aldrich	Cat#X100-1L
Experimental models: Cell lines		
Human cervical carcinoma cell line ME-180	ATCC	HTB-33
Human promyelocytic leukemia cell line HL-60	ATCC	CCL-240
Recombinant DNA		
pAMJ2630 vector (modified pBR322 expression vector)	Bioneer A/S, Denmark	N/A
Software and algorithms		
Prism version 9.1.0–9.1.2	GraphPad	https://www.graphpad.com/scientific-software/prism/
FlowJo software version 10.7	FlowJo LLC	https://www.flowjo.com/
Other		
Sartorius™ Sartopore™ 2 150 Membrane Filter Capsule	Fisher Scientific	Cat#15775093
ELISA plate	Corning Costar, High Binding	Cat#3369
96-well Round bottom culture plates	Corning Costar, High Binding	Cat#3799
24-well tissue culture plates	Thermo Scientific	Cat#142475
Gibco X10 RPMI 1640 medium (1X), liquid with L-glutamine	Gibco	Cat#21875091
Dulbecco's Modified Eagle Medium (DMEM)	HyClone	Cat#SH300.22.01
Gibco™ Penicillin-Streptomycin (10,000 U/mL)	Gibco	Cat#15140-122
GlutaMax-1 (100X)	Gibco	Cat#35050038
Gelatin	Sigma-Aldrich	Cat#G9391-100G
Gibco™ HBSS (10X), no calcium, no magnesium, no phenol red	Gibco	Cat#14185-052
Gibco™ HBSS (10X), calcium, magnesium, no phenol red	Gibco	Cat#14065056
Bovine Serum (FetalClone I, for HL60 cells)	HyClone	Cat#SH30080.03
Fetal Bovine Serum (defined FBS, for OB)	HyClone	Cat#SH30070.03
Fetal Bovine Serum	Sigma-Aldrich	Cat#F7524
Todd-Hewitt-yeast extract (THY) broth	Substratavdelningen, Lund University	N/A
Todd-Hewitt-yeast extract (THY-agar)	Substratavdelningen, Lund University	N/A

RESOURCE AVAILABILITY

Lead contact

Further information and requests for resources and reagents should be directed to and will be fulfilled by the lead contact, Bengt Johansson-Lindbom (bengt.johansson_lindbom@med.lu.se)

Materials availability

All unique/stable reagents generated in this study are available from the Lead Contact with a completed Materials Transfer Agreement. Please direct resource and reagent requests to the Lead Contact specified above, Bengt Johansson-Lindbom.

Data and code availability

- All data reported in this paper (de-identified) will be shared by the lead contact upon request
- This paper does not report original code
- Any additional information required to reanalyze the data reported in this paper is available from the lead contact upon request.

EXPERIMENTAL MODEL AND SUBJECT DETAILS

Human subjects

Design of the clinical phase I study of the prototype vaccine GBS-NN

The current investigation was conducted with serum samples from a two-part, randomized, double blind, placebo-controlled study conducted in a total of 240 healthy adult non-pregnant women who were required to use an effective form of contraception throughout the active phase of the trial (NCT02459262). The complete study design, including description of participants, randomizing and masking, and procedures, has been described elsewhere.[10]

Study of naturally acquired αC-N and Rib-N specific Abs in paired pregnant women and neonates

The study was based on serum samples from pregnant women, admitted to the maternity ward, department of obstetrics, Rigshospitalet, Copenhagen, Denmark, during January 28 to August 8, 2016, and paired serum samples from cord and neonatal blood. Maternal venous blood and cord blood samples were collected on the day of delivery (n = 152). Median gestational age was 40 weeks (95% CI 40–41 weeks, range 36–42 weeks). Maternal and neonatal venous blood samples were then collected from the same cohort approximately one month (median 37 days, 95% CI 33–42 days, n = 105 maternal/neonatal pairs) and two months (median 74 days, 95% CI 71–80 days, n = 61 maternal/neonatal pairs) after birth. The study was approved by the National Committee on Health Research Ethics in Denmark (Jr no.: H-15008888) and by the Danish Data Protection Agency (RH-2015-63, I-Suite no: 03781). Parents gave oral and written informed consent before enrollment. The initial contact with potential participants took place at the midwifery consultation in connection with the routine antenatal check-up at gestation week 36–38, where the women/couples were approached by the project midwife.

Bacterial strains

GBS strains A909 (αC/CPS type Ia), BM110 (Rib/CPS type III), NCTC12906 (Alp1/CPS type Ia) and NEM316 (Alp2/CPS type III) were kindly provided by Tomas Areschoug (Department of Laboratory Medicine, Lund University, Sweden). Clinical GBS isolates collected from cases of invasive disease in Johannesburg, South Africa, during 2012–2014 have previously been described.[34] PCR based typing of Alp genes was done by SSI, Copenhagen, Denmark, according to established protocols.[55]

Cell lines

The human promyelocytic leukemia cell line HL-60 (ATCC, CCL-240) was cultured in RPMI 1640 medium (Gibco, 21875091) supplemented with 10% of heat-inactivated FetalClone I serum (HyClone, SH30080.03), GlutaMax (Gibco, 35050038), and Penicillin-Streptomycin (Gibco, 15140-122) at 37°C in 5% CO_2. The cells were maintained at a density lower than 10^6 viable cells/mL by passaging with fresh medium at least two times per week.

The human cervical carcinoma cell line ME180 (ATCC HTB-33) was cultured in Dulbecco's Modified Eagle Medium (DMEM; HyClone SH300.22.01) containing 10% Fetal Bovine Serum (FBS; Sigma, F7524), penicillin and streptomycin, at 37°C in 5% CO_2. For passage (~twice a week), the cells were treated with trypsin-EDTA for 5 min at 37°C and reseeded in fresh medium.

METHOD DETAILS

Cloning and production of recombinant Alp-N proteins

Recombinant Alp-N domains were produced by Bioneer A/S, Denmark, at the commission of MinervaX (Denmark). Codon-optimized genes encoding the four antigens αC-N, Rib-N, Alp1-N and Alp2/3-N were synthesized by GeneArt (Thermo Fisher Scientific) and inserted into the unique *Nde*I and *Xho*I restriction sites present in a modified pBR322-based expres-

sion vector, pAMJ2630, under the control of the IPTG inducible *lac* promoter (E-c vector pAMJ2630 developed by Bioneer A/S), and transformed into competent *E. coli* BL21 cells (Novagen) using standard cloning and transformation procedures. The cloning junctions were confirmed by DNA sequencing. The pAMJ2630 expression vector encodes resistance to kanamycin. Recombinant BL21 strains transformed with the individual expression vectors were grown in 1L bioreactors in 800 mL medium supplemented with 25 mg/L kanamycin sulfate at 37°C and with stirring. Cultures were induced at an OD600 between 4 and 5 by addition of 0.1 mM IPTG and harvested 4 hours later by centrifugation. Cell suspensions were re-suspended in ice-cold lysis buffer (10 mM Tris-HCl, pH 8.0 containing 40 units/mL of Benzonase®) corresponding to an OD_{600} about 150. The cells were lysed by one pass through a high-pressure homogenizer (Constant TS 1,1 kW, Constant Systems Limited, UK) at 1500 bar with cooling at 5°C. 50 mL of lysis buffer (without Benzonase®) was passed through the equipment after each homogenization run and mixed with the cell lysate. After homogenization the cell debris was removed by centrifugation (20,000 × g for 60 min at 4 °C) and supernatants containing the Alp-N antigens were sterile filtered (Sartopore 2 150) and then frozen at −20°C until purification.

Purification of recombinant Alp-N proteins

αC-N

The cell lysate supernatant was thawed, and pH adjusted to 4.0 using 1M HCl. Precipitated *E. coli* proteins were removed by centrifugation at 20.000 xg for 15 min at +10°C. The supernatant was diluted to a conductivity of 3 mS/cm with water. αC-N was then purified on a 50 mL Capto S ImpAct column equilibrated with 20 mM sodium acetate, pH 4.0, and eluted using a linear 0 to 0.4 M NaCl gradient over 15 CV. Fractions containing αC-N were pooled, pH adjusted to 6.5 with 0.5 M NaOH, concentrated, and buffer exchanged into 20 mM Bis-Tris/HCl, pH 6.5 using a stirred Amicon ultrafiltration cell with a 3 kDa Ultracel membrane. αC-N was then further purified on a 58 mL Capto Q ImpRes column equilibrated with 20 mM Bis-Tris/HCl, pH 6.5, and eluted using a linear 0 to 0.3 M NaCl gradient over 18 CV. Fractions containing AlphaC-N were pooled and concentrated to approximately 1 mg/mL by ultrafiltration and finally buffer exchanged into PBS pH 7.4 (Gibco) on a HiPrep 26/10 desalting column. The protein solution was aliquoted and stored at −20°C.

Rib-N

Rib-N was purified as described above for αC-N with the following modifications. *E. coli* protein precipitation and cation exchange were done at pH 4.5 and the anion exchange was done in 20 mM Tris/HCl, pH 8.0.

Alp1-N and Alp2/3-N

The cell lysate supernatant was thawed, and DNA precipitated by adding streptomycin sulphate to 1.5 % (w/v) and 1 h incubation at +4°C followed by centrifugation at 20,000 g for 20 min at +10°C. Then pH was adjusted to 4.0 using 1M HCl and precipitated protein was removed by centrifugation at 20,000 g for 15 min at +10°C. The protein solution was then desalted into 20 mM sodium acetate, pH 4.0 on a HiPrep 26/10 desalting column. The Alp-N protein was then purified on a Capto S ImpAct column, buffer exchanged into 20 mM Bis-Tris/HCl, pH 6.5, by ultrafiltration and further purified on Capto Q ImpRes column, as described above. Finally, Alp-N protein was concentrated to 1 mg/mL, buffer exchanged into PBS, pH 7.4, and stored, as described above.

All proteins were > 95% pure as judged by SDS-PAGE.

Quantitation of Alp-N-specific IgG, IgG1, IgG2 and IgA *in sera from the study subjects*

Quantification of IgG, IgG1, IgG2 and IgA Abs against αC-N, Rib-N, Alp1-N and Alp2/3-N by ELISA was done as recently described for quantification of Abs against the intact GBS-NN protein.[10] Calibrated human immunoglobulin for subcutaneous administration (Subcuvia, 160 mg/mL, Baxalta, Sweden) (SCIG) was used a standard with known concentration of Abs specific to each Alp-N protein. The SCIG preparation was calibrated for all isotypes and IgG subclasses against all individual Alp-N domains based on equivalence of absorbance between a reference ELISA and the anti-Alp-N ELISA performed in parallel under identical conditions, as described.[10] For coating, ELISA plate (Corning Costar, High Binding) were incubated overnight at 4°C with 100 μl of relevant recombinant Alp-N domain per well, all diluted to 0.5 μg/mL in PBS. Pre- and post-vaccination serum from the study subjects was serially diluted in PBS-2% BSA-0.05% Tween 20 (sample buffer) and added to the wells (100 μl/well). Calibrated SCIG was serially diluted in sample buffer and added to separate rows of wells on each plate (100 μl/well) as a standard. After 2 h incubation at room temperature the plate was washed and horseradish peroxidase (HRP)-conjugated detection Abs diluted in the sample buffer were added to all wells (100 μl/well): goat anti-human IgG (γ-chain) $F(ab')_2$-HRP (Sigma A2290), goat anti-human IgA (α-chain)-HRP (Life Technologies A18787), goat anti-human IgM (μ-chain) $F(ab')_2$-HRP (Sigma A4290), mouse anti-human IgG1 Fc-HRP (Life Technologies MH1715) or mouse anti-human IgG2 Fc-HRP (Life Technologies MH1722). The plate was incubated with HRP-conjugated detection Abs for 1 h at room temperature. After washing, HRP was detected using tetrametylbenzidine (TMB; Sigma T8768)-hydrogen peroxide (100 μl/well). Color reaction was stopped with 50 μl of 1 M sulfuric acid/well and the absorbance was read in SpectroStar photometer at 450 nm wavelength. Raw data were analyzed using four parameters logistic (4PL) fit function in Prism7 software (GraphPad). Concentrations of Abs in serum samples were computed for all sample dilutions using absorbance values of calibrated SCIG dilutions as standard curves. Linearity of serum sample curves was assessed by computing coefficient of variation (CV) for all sample dilutions. When the CV for at least 3 dilutions was < 20% the result was considered valid.

Quantitation of Alp-N specific IgM in sera from the study subjects

As the SCIG preparation does not contain IgM Abs, Alp-N specific IgM concentrations in the serum samples were determined by equivalence of absorbance between a reference IgM capture ELISA and the anti-Alp-N ELISA performed in parallel for each individual plate under identical conditions. Part of the 96-well microtiter plate (Corning Costar, High Binding) was coated with 100 μl/well of goat anti-human IgM (H+L) $F(ab')_2$ (Sigma, SAB3701393) at 1 μg/mL in PBS. Another part of the same plate was coated with 100 μl/well of recombinant Alp-N domain, all at 0.5 μg/mL in PBS. Several wells were left uncoated (negative control). After overnight incubation at 4°C the plate was washed with PBS-0.05% Tween 20. Purified human serum IgM (InVitrogen 31146) was serially diluted in sample buffer, and 100 μl/well of serial dilutions was added to the wells coated with the capture Ab. Serum samples were serially diluted in the sample buffer and added (100 μl/well) to the wells coated with Alp-N proteins. After 2 h incubation at room temperature the plate was washed and goat anti-human IgM (μ-chain) $F(ab')_2$-HRP (Sigma A4290) was added to all wells. The plate was incubated with the HRP-conjugated detection Ab for 1 hour at ambient temperature. After washing, the plates were developed and read as described above. Raw data were analyzed using the 4PL fit function in Prism7 software (GraphPad). Absorbance values of the purified human IgM standard in the reference ELISA were compared with absorbance values obtained for serially diluted serum samples. Alp-N specific IgM concentrations (μg/mL) were assigned to the serum samples, based on equal absorbance of the known reference IgM concentration and serum dilutions derived from parallel parts of both curves.

Opsonophagocytic killing assay (OPkA)

OPkA was performed as described by Nahm and Burton for anti-capsular Abs.[35] In brief, HL-60 cells were harvested for differentiation twice a week and differentiated into neutrophils in RPMI 1640 medium supplemented with 10% heat-inactivated FetalClone I serum and 0.8% dimethylformamide (DMF) (Sigma, 227056) for 5 days at 37°C in 5% CO_2. Typically, after 20 passages the HL-60 cells became refractory to DMF-induced differentiation, as indicated by decrease of the CD35 and increase of the CD71 cell surface markers, and a new HL-60 culture was initiated.

A909 and BM110 GBS strains were grown in Todd-Hewitt-yeast extract (THY) broth at 37°C. Bacteria were harvested at mid-log growth phase after reaching optical density (OD_{600}) of approximately 0.6. Bacteria were aliquoted in the growth medium containing 16% glycerol, and frozen at -80°C. Colony forming units (CFU) in frozen bacteria stocks were enumerated in thawed aliquots in the OPkA format in the presence of 12.5% baby rabbit complement (BRC; Pel-Freez, Arkansas) and absence of serum, as described by Nahm and Burton.[35]

Pre-immune and post-vaccination sera from the study subjects were heat-inactivated at 56°C for 30 min and serially diluted in 3-fold steps from 10- to 21,870-fold in 96-well round bottom culture plates (Corning Costar 3799), using opsonization buffer (OB; 0.95% Hanks' balanced salt solution containing Ca^{2+} and Mg^{2+} ions, 0.1% gelatin, and 5% heat inactivated Fetal Bovine Serum). Thawed bacteria were added (1000 CFU/well), and the plates were agitated on an orbital shaker (700 rpm) for 30 minutes at room temperature. Thereafter, differentiated HL-60 cells were mixed with the BRC and added to the wells to a final concentration of 12.5% BRC and a bacteria:cell ratio of 1:400. The plates were incubated on an orbital shaker (700 rpm) at 37°C in 5% CO_2 for 45 min. Next, the plates were placed on ice to stop phagocytosis. After 20 min, 5 μl aliquots of the mixture were inoculated onto rectangular 25 x 25 cm THY-agar plates. Following overnight incubation of the plates at 37°C and 5% CO_2, CFU were counted. Reciprocal of serum dilution resulting in 50% killing of bacteria was calculated using maximum CFU numbers from wells without serum (absolute OPkA titer). Vaccination-induced OPkA titer (ΔOPkA) was calculated as a difference between OPkA titers of pre-immune and vaccine-induced OPkA titers. Killing curves and OPkA titers were generated in Prism 7 software.

Inhibition of serum OPkA activity by GBS-NN

To assess participation of Alp-N-specific Abs in opsonophagocytic activity of sera from the study subjects, pre-immune and post-vaccination sera were pre-incubated with GBS-NN. Ten-fold diluted sera in OB containing 0 or 200 ng/mL of GBS-NN were incubated for 1 h at ambient temperature. Following this absorption step the sera +/- GBS-NN were diluted at 3-fold steps from 10- to 21,870-fold in 96-well, round bottom, plates, and the assay commenced as described above. Percent inhibition of killing by GBS-NN was calculated using a formula: (50% killing titer of non-adsorbed serum – 50% killing titer of adsorbed serum) / 50% killing titer of non-adsorbed serum X 100.

Assessment of binding of Alp-N-specific serum IgG to whole GBS by flow cytometry (FACS)

A909 and BM110 GBS strains were cultured in 45 ml of THY broth each until they reached mid-log growth phase (OD ≈ 0.6 at 600 nm wavelength). Bacteria were pelleted at 2200 rcf for 10 min, washed with PBS, and resuspended in PBS containing 20% glycerol at a density resulting in OD=2.5 (approximately 5 x 10^8 CFU/mL). Bacterial suspensions were aliquoted and stored at -80°C.

For FACS analysis the pre-immune and post-vaccination sera were serially diluted in FACS buffer (PBS-2% FBS-2mM EDTA). Thawed bacteria were pelleted and resuspended in FACS buffer to an OD=1 (approximately 2 x 10^8 CFU/mL). Serially diluted sera were added to the bacterial suspension and incubated for 30 min at ambient temperature with constant agitation. After washing, bound Abs were detected by a fluorescent labelled secondary Ab, followed by fixation of the bacteria using 2% paraformaldehyde. Samples were acquired on a BD Accuri flow cytometer. FACS results were analyzed in FlowJo 10.1. The population of GBS could be gated between FSC-H 10^4-10^6 and SSC-H 10^3-10^5 with some variation between strains, likely due to differences in chain length.

Geometric mean fluorescence intensity (GMFI) of the whole GBS population was reported to minimize the effect of chain length variation between samples.

Epithelial cell invasion inhibition assay

The assay was a modified procedure of Stålhammar-Carlemalm et al.[11] Human cervical carcinoma cell line ME180 (ATCC HTB-33) was cultured in 24-well tissue culture plates (Thermo Scientific 142475) in 500 μl/well of Dulbecco's Modified Eagle Medium (DMEM; HyClone SH300.22.01) containing 10% Fetal Bovine Serum (FBS; Sigma F7524), penicillin and streptomycin, at 37°C in 5% CO_2. ME180 cells were cultured to approximately 80% confluence on the day of the assay. At this stage the cells reached a density of approximately 0.6 x 10^6/well and were >90% viable, as indicated by counts of cells detached from control wells by trypsin-EDTA and stained with Trypan Blue.

A909 and BM110 GBS strains were cultivated in THY broth overnight at 37°C in normal atmosphere. Bacteria were pelleted at 2200 rcf for 5 min, resuspended in 10 ml of PBS, and OD_{600} of the suspension was recorded. Number of CFU corresponding to a given OD value was estimated prior to the assay using A909 and BM110 GBS cultures grown under identical conditions. Bacteria were diluted with DMEM to an OD value corresponding to 2 x 10^6 CFU/mL. Aliquots of the bacterial suspension were inoculated onto blood-agar to verify actual infection dose by CFU counts. Pre-immune and post-vaccination sera from the study subjects were then added to the bacterial suspension in amounts that resulted in 1 ng/mL, 10 ng/mL, 50 ng/mL, and 100 ng/mL of homologous Alp-N-specific IgG plus IgA in the final mixture. Hyper-immune serum from a rabbit immunized with GBS-NN mixed with bacteria was used as a positive control (100% inhibition of invasion). Bacteria suspension without serum was used as negative control (100% invasion). Bacteria +/- serum mixtures were agitated on an orbital shaker (300 rpm) at ambient temperature.

The ME180 cell culture medium was replaced with DMEM without FBS and antibiotics and the cell culture was incubated by 1h at 37°C with 5% CO_2. Next, the medium was removed and 500 μl of bacteria suspension (1 x 10^6 CFU) +/- serum from the study subjects was added to the wells. Bacteria plus rabbit serum and bacteria without serum were added to control wells. The plate was centrifuged at 800 rcf to facilitate contact between bacteria and the cell layer and incubated for 1 h at 37°C in 5% CO_2. Next, the plate was washed with PBS, and DMEM medium containing gentamycin and penicillin was added to kill extracellular bacteria. After 2 h incubation, cell layers were washed with PBS and the cells were lysed with 1 ml of ice-cold 0.025% Triton X-100. Serial dilutions of the lysates were plated on blood-agar plates (4 plates/well of cell culture plate). CFU were counted after overnight incubation at 37°C in 5% CO_2. Raw data were processed in Microsoft Excel 14.5. The fraction of bacteria invading ME180 cells in the absence of serum was 0.1%–0.4% of the inoculum. Mean CFU values from wells with and without serum were used to compute percentage of invasion inhibition mediated by serum Ab specific to Alp-N proteins.

QUANTIFICATION AND STATISTICAL ANALYSIS

All statistical analyses were performed using GraphPad Prism version 9.1.0–9.1.2 for Mac, GraphPad Software, San Diego, California USA. One-way ANOVA or paired two-tailed t test was used on logarithmically transformed values as indicated to compare the differences between Ab concentrations in pre-immune and post-vaccination sera. Pearson correlations (of logarithmically transformed values) was used to test the strength of the association between pre- and post-vaccination serum Ab concentrations, between serum Ab concentrations and OPkA titers, and between Ab concentrations and intensity of Ab binding to whole GBS in FACS.

ADDITIONAL RESOURCES

Clinical trial number: NCT02459262.

Cell Reports Medicine, Volume *3*

Supplemental information

A group B *Streptococcus* alpha-like protein subunit vaccine induces functionally active antibodies in humans targeting homotypic and heterotypic strains

Andrzej Pawlowski, Jonas Lannergård, Majela Gonzalez-Miro, Duojia Cao, Sara Larsson, Jenny J. Persson, Geoff Kitson, Michael Darsley, Ane Lilleøre Rom, Morten Hedegaard, Per B. Fischer, and Bengt Johansson-Lindbom

Table S1. Alp-N domain specific serum IgG, IgA and IgM geometric mean concentrations (GMC) and fold-increases relative pre-vaccination baseline levels (Day 0) four weeks after the second vaccine dose (Day 57). The two separate cohorts receiving 50 µg GBS-NN plus AlOH in the clinical phase IA (n=8) and 1B (n=45) studies are included in the analyses (n=53 in total). Related to Figures 1 and 2.

	**IgG GMC**			**IgA GMC**			**IgM GMC**		
	(µg/ml) (95% CI)		Fold-increase	(µg/ml) (95% CI)		Fold-increase	(µg/ml) (95% CI)		Fold-increase
	Day 0	Day 57	(95% CI)	Day 0	Day 57	(95% CI)	Day 0	Day 57	(95% CI)
αC-N	**0.12**	**5.45**	**44**	**0.15**	**2.23**	**15**	**0.89**	**1.65**	**1.9**
	(0.09 - 0.16)	(3.71 - 8.01)	(32 - 62)	(0.11 - 0.21)	(1.35 - 3.69)	(10 - 21)	(0.70 - 1.13)	(1.34 - 2.04)	(1.4 - 2.4)
Rib-N	**0.05**	**2.22**	**42**	**0.09**	**0.91**	**10**	**1.16**	**1.42**	**1.2**
	(0.04 - 0.06)	(1.67 - 2.96)	(31 - 57)	(0.07 - 0.11)	(0.61 - 1.35)	(7.6 - 14)	(0.96 - 1.38)	(1.12 - 1.80)	(1.0 - 1.5)
Alp1-N	**0.14**	**1.15**	**8.1**	**0.17**	**0.56**	**3.4**	nd	nd	-
	(0.11 - 0.18)	(0.71 - 1.84)	(5.8 - 11)	(0.12 - 0.22)	(0.35 - 0.89)	(2.5 - 4.6)	-	-	-
Alp2/3-N	**0.21**	**2.42**	**11**	**0.13**	**1.04**	**8.1**	nd	nd	-
	(0.16 - 0.28)	(1.52 - 3.86)	(8.0 - 16)	(0.09 - 0.17)	(0.66 - 1.64)	(6.0 - 11)	-	-	-

Table S2. Vaccine-induced OPk (ΔOPkA titers) of clinical isolates possessing the αC gene and collected from cases of EOD (reference 34) as determined with pre- and post-vaccination sera from two vaccinees achieving high levels of αC-specific IgG. Related to Figure 4.

Serum pair[a]	GBS isolate	Invasive disease	Capsule type	ΔOPkA titer[d]	TR A909[e]
A03[b]	1001	EOD	Ia	2050	1.93
	1044	EOD	V	3430	3.27
	1047	EOD	Ib	1900	n.d.
	1084	EOD	II	3630	6.05
B10[c]	1001	EOD	Ia	6015	1.99
	1044	EOD	V	8880	3.36
	1047	EOD	Ib	16700	n.d.
	1084	EOD	II	10925	7.09

[a] day 0 and day 57 post vaccination sera
[b] αC-specific IgG conc. were 0.37 and 42 µg/ml for day 0 and day 57, respectively
[c] αC-specific IgG conc. were 0.67 and 93 µg/ml for day 0 and day 57, respectively
[d] vaccine-induced OPkA titer = titer (day 57) - titer (day 0)
[e] Relative titer = ΔOPkA titer for isolate / ΔOPkA titer for strain A909
n.d. ΔOPkA titer for strain A909 not determined in experiment

Table S3. Pre- and post-vaccination (day 0 versus day 57) concentration of antibodies against αC-N and Rib-N, as well as vaccine-induced OPk titers (ΔOPkA titer) against A909 and BM110, for the selected sera studied in Figure 5A. Related to Figure 5. For results presented in Figure 5A, all sera were assayed at a fixed dilution of 1/100.

Subject	αC-N IgG (µg/ml)		αC-N IgA (µg/ml)		Rib-N IgG (µg/ml)		Rib-N IgA (µg/ml)		ΔOPkA titer	
	Day 0	Day 57	Day 0	Day 57	Day 0	Day 57	Day 0	Day 57	A909	BM110
A02	0.10	4.85	0.10	3.82	0.09	5.33	0.12	1.99	1100	380
A03	0.37	42.4	1.16	36.9	0.04	5.61	0.16	2.80	8445	210
A06	0.16	42.0	0.08	10.8	0.02	2.47	0.08	1.73	9690	45
A08	0.21	27.0	1.31	15.3	0.04	7.54	0.12	2.02	5880	310
